# RICE ACYL-COA-BINDING PROTEIN6 Affects Acyl-CoA Homeostasis and Growth in Rice

**DOI:** 10.1186/s12284-020-00435-y

**Published:** 2020-11-06

**Authors:** Wei Meng, Lijian Xu, Zhi-Yan Du, Fang Wang, Rui Zhang, Xingshun Song, Sin Man Lam, Guanghou Shui, Yuhua Li, Mee-Len Chye

**Affiliations:** 1 Key Laboratory of Saline-alkali Vegetation Ecology Restoration (Northeast Forestry University), Ministry of Education, Harbin, 150040 China; 2 College of Life Science, Northeast Forestry University, Harbin, 150040 China; 3 College of Advanced Agriculture and Ecological Environment, Heilongjiang University, Harbin, 150080 China; 4 Department of Biosciences and Bioengineering, University of Hawaii at Manoa, Honolulu, HI 96822 USA; 5 School of Biological Sciences, The University of Hong Kong, Pokfulam, Hong Kong; 6 State Key Laboratory of Molecular Developmental Biology, Institute of Genetics and Developmental Biology, Chinese Academy of Sciences, Beijing, China; 7 Lipidall Technologies Company Limited, Changzhou, 213000 China

**Keywords:** Acyl-CoA esters, Acyl-CoA-binding protein, Jasmonic acid, Lipid metabolism, Peroxidases, Reactive oxygen species

## Abstract

**Backgrounds:**

Acyl-coenzyme A (CoA) esters are important intermediates in lipid metabolism with regulatory properties. Acyl-CoA-binding proteins bind and transport acyl-CoAs to fulfill these functions. RICE ACYL-COA-BINDING PROTEIN6 (OsACBP6) is currently the only one peroxisome-localized plant ACBP that has been proposed to be involved in *β*-oxidation in transgenic Arabidopsis. The role of the peroxisomal ACBP (OsACBP6) in rice (*Oryza sativa*) was investigated.

**Results:**

Here, we report on the function of OsACBP6 in rice. The *osacbp6* mutant showed diminished growth with reduction in root meristem activity and leaf growth. Acyl-CoA profiling and lipidomic analysis revealed an increase in acyl-CoA content and a slight triacylglycerol accumulation caused by the loss of OsACBP6. Comparative transcriptomic analysis discerned the biological processes arising from the loss of *OsACBP6*. Reduced response to oxidative stress was represented by a decline in gene expression of a group of peroxidases and peroxidase activities. An elevation in hydrogen peroxide was observed in both roots and shoots/leaves of *osacbp6*. Taken together, loss of OsACBP6 not only resulted in a disruption of the acyl-CoA homeostasis but also peroxidase-dependent reactive oxygen species (ROS) homeostasis. In contrast, *osacbp6-*complemented transgenic rice displayed similar phenotype to the wild type rice, supporting a role for OsACBP6 in the maintenance of the acyl-CoA pool and ROS homeostasis. Furthermore, quantification of plant hormones supported the findings observed in the transcriptome and an increase in jasmonic acid level occurred in *osacbp6*.

**Conclusions:**

In summary, OsACBP6 appears to be required for the efficient utilization of acyl-CoAs. Disruption of OsACBP6 compromises growth and led to provoked defense response, suggesting a correlation of enhanced acyl-CoAs content with defense responses.

**Supplementary Information:**

The online version contains supplementary material available at 10.1186/s12284-020-00435-y.

## Background

The primary role of acyl-coenzyme A (CoA) esters is to act as intermediates in lipid synthesis and breakdown (Neess et al. 2015). De novo synthesized fatty acids in the chloroplasts are transferred in the form of C16:0-CoA and C18:1-CoA to the endoplasmic reticulum (ER) for phospholipid and triacylglycerol (TAG) synthesis (Li-Beisson et al. 2013). In seeds following imbibition, TAG is rapidly mobilized to support successful germination and seedling establishment via a series of catabolic pathways (Graham 2008; Li-Beisson et al. 2013). Rapid TAG turnover also occurred in vegetative tissues (Kelly et al. 2013). First, TAG is hydrolyzed by lipases to free fatty acids, followed by activation to CoA esters (Li-Beisson et al. 2013). Subsequently, fatty acyl-CoAs are imported into the peroxisomes by the peroxisomal ATP-binding cassette (ABC) transporter protein COMATOSE (CTS)/PEROXISOME DEFECTIVE 3 (PED3)/PEROXISOMAL ABC TRANSPORTER 1 (PXA1), which possesses thioesterase activity that hydrolyzes fatty acyl-CoAs to free fatty acids and CoA as part of the transport process (De Marcos et al. 2013). Once inside the peroxisome, the formation of long-chain acyl-CoA esters is catalyzed by long-chain acyl-CoA synthetase (Li-Beisson et al. 2013). Following a series of enzymatic *β*-oxidation reactions by acyl-CoA oxidases, multifunctional proteins, and thiolases, fatty acids are degraded to acetyl-CoA for further use in gluconeogenesis and respiration (Li-Beisson et al. 2013).

The intrinsic nature of acyl-CoA is amphipathic (Neess et al. 2015). Although the cellular concentration of acyl-CoAs ranges from several μM to several hundred μM, the concentration of free unbound acyl-CoAs is unknown or could be extremely low (Neess et al. 2015). Usually, acyl-CoAs are bound to membrane lipids or proteins with high affinities, so that the concentration is strictly controlled to below the critical micelle concentration to avoid detergent effects that disrupt the membrane (Neess et al. 2015). The small (10-kD) acyl-CoA-binding proteins (ACBPs) are known to bind acyl-CoA esters (Knudsen et al. 2000). Each ACBP has a conserved acyl-CoA-binding domain that allows the ACBPs to bind acyl-CoA esters, which can then form a cellular acyl-CoA pool and protect acyl-CoAs against hydrolysis (Du et al. 2016). Considering that ACBPs bind acyl-CoAs with high affinities and broad ligand selectivity (C12-C26), ACBPs are believed to be the predominant acyl-CoA carriers/transporters (Knudsen et al. 2000; Neess et al. 2015; Lung and Chye 2016a, b; Guo et al. 2017).

In plants, ACBPs are highly conserved throughout the evolution of land plants and are divided into four classes (Classes I-IV) based on domain architecture (Meng et al. 2011). The functions of Arabidopsis ACBPs (AtACBPs) in development and stress responses have been extensively investigated (Du et al. 2016; Lung and Chye 2016b, 2019). Much evidence indicates that AtACBPs mediate multiple aspects of plant physiology by regulating lipid metabolism in Arabidopsis (Du et al. 2016; Lung and Chye 2016b, 2019). There is only a single 10-kD Class I ACBP member in Arabidopsis, namely AtACBP6, and AtACBP6 has been reported to be a cytosolic protein (Chen et al. 2008). AtACBP1 and AtACBP2 are Class II paralogues of each other and have been proposed to function in the ER in acyl-CoA pool formation and acyl-CoA/lipid trafficking between the plasma membrane and the ER during early embryogenesis (Chen et al. 2010). Furthermore, the overexpression of AtACBP1 in Arabidopsis resulted in the accumulation of phosphatidic acid (PA) during seed germination and seedling development following abscisic acid (ABA) treatment (Du et al. 2013). Similar PA accumulation in AtACBP1 overexpressors occurred during freezing treatment (Du et al. 2010). The interaction between AtACBP1 and phospholipase Dα1 (PLDα1), a phospholipase that generates PA, played an essential role in both studies (Du et al. 2010; Du et al. 2013). In addition, the interaction between AtACBP1 and STEROL C4-METHYL OXIDASE1 (SMO1) regulated both fatty acid and sterol homeostasis, and in turn modulated the expression of homeodomain-leucine zipper IV transcription factors (TFs) (Lung et al. 2018). The overexpression of another member, Class III AtACBP3, accelerated leaf senescence increasing the acyl-CoA pool and phosphatidylethanolamine (PE) content (Xiao et al. 2010). The variation in AtACBP3 level and PE composition between AtACBP3-overexpressors and AtACBP3-knockout mutants indicated that AtACBP3 binds and regulates PE in vivo (Xiao et al. 2010). Furthermore, the recombinant proteins of the three cytosolic AtACBPs, comprising Class IV members (AtACBP4 and AtACBP5), and Class I AtACBP6, were reported to bind C16:0-CoA, C18:1-CoA, C18:2-CoA, and C18:3-CoA with different affinities (Hsiao et al. 2014a). The double mutants and the triple mutant of these three cytosolic AtACBPs were altered in acyl-CoA composition and displayed high ABA sensitivity during seed germination (Hsiao et al. 2014a). The decrease in oil bodies and decline in germination rate of *acbp4acbp5acbp6* pollen suggested important roles for the cytosolic AtACBPs in pollen lipid metabolism (Hsiao et al. 2015).

Thus far, the functions of plant ACBPs have been widely elucidated in the eudicots, such as Arabidopsis and *Brassica napus*, although there have been many reports on the characterization of ACBP family members from other plant species, such as *Oryza sativa*, *Agave americana*, *Vernicia fordii*, *Vitis vinifera*, *Helianthus annuus*, *Gossypium hirsutum*, *Jatropha curcas*, and *Elaeis guineensis* (Du et al. 2016; Raboanatahiry et al. 2018; Liao et al. 2019; Amiruddin et al. 2020). Other than Arabidopsis and *Brassica napus*, the functions of ACBPs from other plant species have been mostly investigated using transgenic Arabidopsis (Takato et al. 2013; Meng et al. 2014; Panthapulakkal Narayanan et al. 2019). Previously, we have reported a peroxisome-localized rice Class IV ACBP, OsACBP6, which is currently the only plant ACBP identified to be localized at the peroxisome (Meng et al. 2014). The overexpression of *OsACBP6* in the Arabidopsis *β*-oxidation-deficient mutant *pxa1* rescued indole-3-butyric acid (IBA) sensitivity and jasmonic acid (JA) production after wound treatment, suggesting that OsACBP6 may be involved in peroxisomal *β*-oxidation (Meng et al. 2014). In this study to investigate the function of the peroxisomal OsACBP6 in rice, we analyzed a T-DNA insertional mutant of OsACBP6 (*osacbp6*) that demonstrated its reduction in root meristem activity and leaf growth. The results from comprehensive acyl-CoA and lipid profiling indicated that disruption of *OsACBP6* compromised the maintenance of the acyl-CoA pool and altered lipid composition in leaves. By comparative transcriptomic analysis of the shoots/leaves and roots from various growth stages, differentially-expressed genes were identified in *osacbp6*. Gene ontology (GO) enrichment analysis and functional classification provided insights into the function of OsACBP6 in the oxidative stress response. Accumulation of reactive oxygen species (ROS) and elevation of the JA was evident in *osacbp6*.

## Results

### The Rice *osacbp6* Mutant Displayed Growth Retardation

**Fig. 1:**
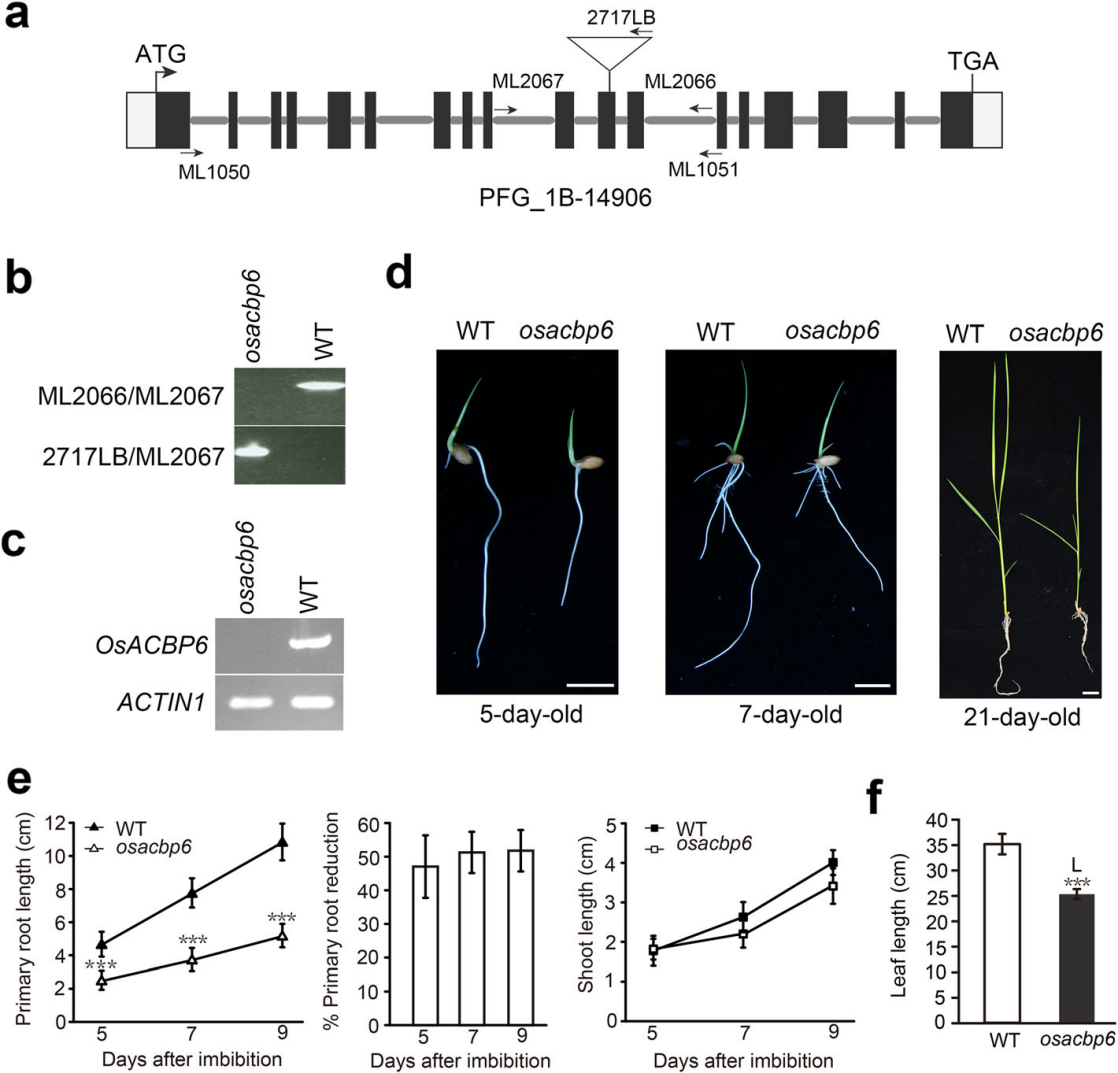
Characterization of the rice *osacbp6* mutant. **a** T-DNA insertion in *OsACBP6*, and primer locations used for PCR genotyping. Black and grey boxes indicate coding and untranslated regions, respectively. **b** PCR genotyping of *osacbp6*. Primer combinations ML2066/ML2067 (top gel) and 2717LB/ML2067 (bottom gel) in PCR to identify *osacbp6* homozygous mutant. **c** Semi-quantitative RT-PCR showing the knockout of *OsACBP6* in *osacbp6*. *ACTIN1* served as a control. **d** Growth of wild type (WT, *Oryza sativa* var. *japonica* cv. Dongjin) and *osacbp6*. Images from left to right represent 5-, 7-day-old seedlings grown in water, and 21-day-old soil-grown seedling. Bars, 1 cm. **e** The primary root length (left) and shoot length (right) comparison between WT and *osacbp6*. Primary root reduction of *osacbp6* is shown in the middle. Values are mean ± SD (*n* = 25). **f** Comparison of the leaf length of 21-day-old soil-grown seedlings between WT and *osacbp6*. Values are mean ± SD (*n* = 5). Asterisks indicate significant differences between WT and *osacbp6* as evaluated by Student’s *t*-tests: ***P < 0.001

DNA sequence analysis of the *osacbp6* mutant indicated that the T-DNA had inserted into the 11th exon of *OsACBP6* as shown in Fig. 1a. The location of the T-DNA insertion in *OsACBP6* was confirmed by PCR using a combination of gene-specific primers, ML2066, and ML2067, as well as T-DNA left border primers 2717LB and ML2067 (Fig. 1b). Semiquantitative RT-PCR using gene-specific primers ML1050 and ML1051 confirmed the absence of *OsACBP6* expression in the mutant (Fig. 1c). The homozygous *osacbp6* line was then used in subsequent experiments.

As OsACBP6 had been proposed to play a role in fatty acid *β*-oxidation through its acyl-CoA binding ability (Meng et al. 2014), *osacbp6* was examined for the typical phenotypes related to defects in *β*-oxidation. When *osacbp6* and the wild type were tested in seed germination and seedling establishment in water, both germinated at about the same time but the development of the roots of *osacbp6* seedlings was repressed with primary root reduction around 40% (Fig. 1d and e). When *osacbp6* and the wild type were grown in soil, *osacbp6* root development was retarded (around 30% primary root reduction), but not as severe as previously observed in water (Fig. S1). In both situations, there was no obvious difference in the length of the shoots between *osacbp6* and the wild type at the early stage of the seedling development (Fig. 1e, Fig. S1b). However, the leaf length of 21-day-old *osacbp6* was significantly shorter than the wild type with a reduction of around 25% (Fig. 1d and f). Furthermore, *osacbp6* grains were reduced in length but remained unchanged in width in comparison to the wild type (Fig. S2a). Lastly, given that the product of IBA *β*-oxidation is indole-3-acetic acid (IAA) which has growth inhibition effect, the IBA sensitivity of *osacbp6* was examined to investigate whether *β*-oxidation process was blocked in *osacbp6*. Reduced primary root elongation of *osacbp6* after IBA treatment indicated that *osacbp6* was sensitive to growth inhibition by IBA (Fig. S2b), suggesting the IBA *β*-oxidation was not blocked in *osacbp6*. Taken together, from phenotypic observations it appears that *β*-oxidation was not severely affected due to the loss in OsACBP6.

**Fig. 2:**
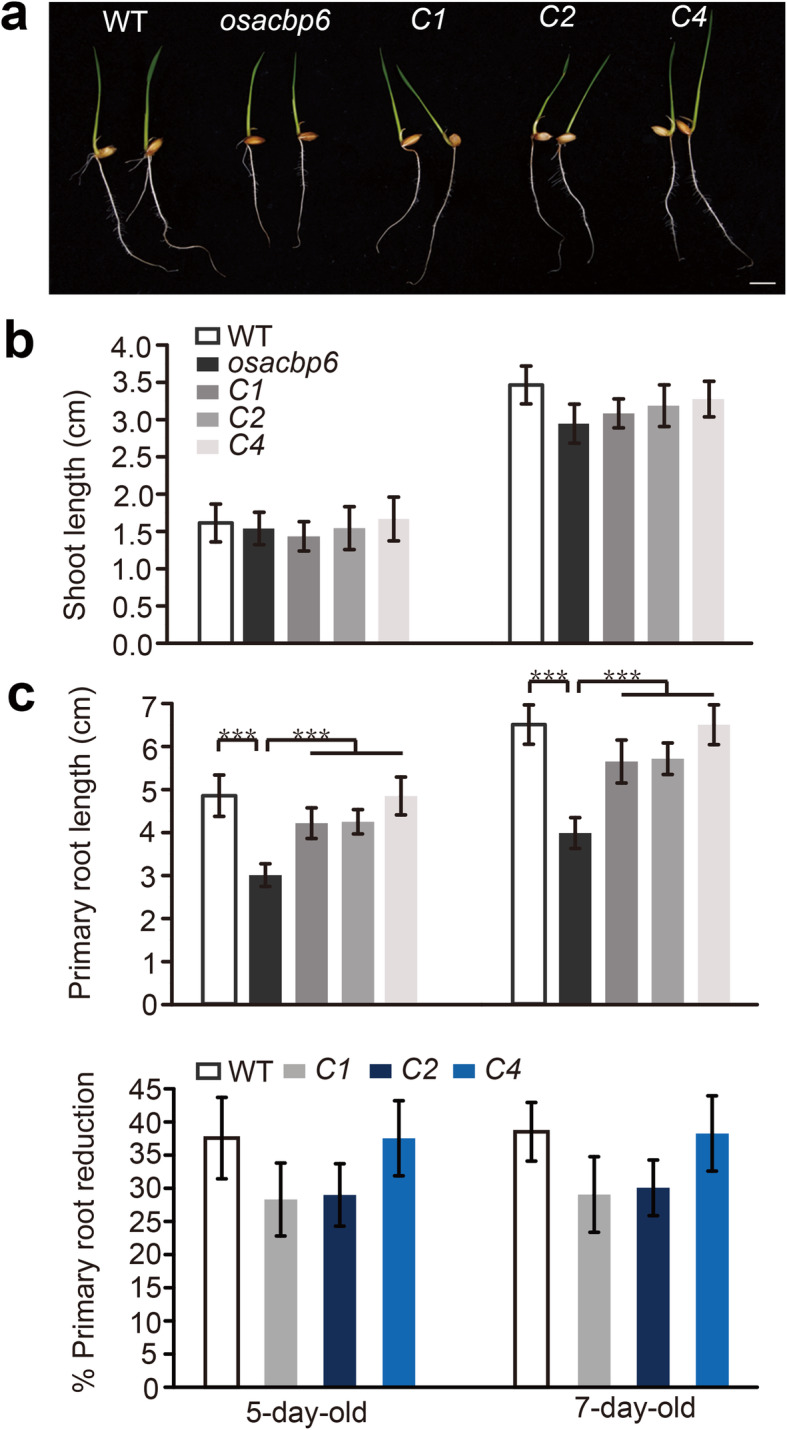
Recovery in growth retardation of the rice *osacbp6* mutant by complementation of *OsACBP6*. **a** Growth of 7-day-old seedlings of wild type (WT), *osacbp6* and complemented lines *osacbp6*-*C1* (*C1*), *osacbp6*-*C2* (*C2*) and *osacbp6*-*C4* (*C4*). Bar, 1 cm. **b** Shoot length of WT, *osacbp6*, *C1*, *C2* and *C4*. Values are means ± SD (*n* = 10). **c** Primary root length of WT, *osacbp6*, *C1*, *C2*, and *C4* (upper panel) and primary root reduction of *osacbp6* relative to WT, *C1*, *C2*, and *C4* (lower panel). Values are means ± SD (*n* = 10). Asterisks indicate significant differences as evaluated by Student’s *t*-tests: ***P < 0.001. WT, *Oryza sativa* var. *japonica* cv. Dongjin

When the *osacbp6* mutant was complemented, the three independent complemented lines (designated as *osacbp6-C1*, *osacbp6-C2*, and *osacbp6-C4*) displayed normal growth similar to the wild type, including the primary root length, 21-day-old leaf length, and grain length (Fig. 2, Fig. S3). Moreover, the phenotype of seedlings from hemizygous complemented line *osacbp6-C4* were examined. In contrast with wild type normal growth of primary root and leaves, seedlings from hemizygous complemented line contained both wild type growth (168) and retarded growth (51) of roots and leaves with a 3:1 segregation ratio (χ^2^ = 0.34). These findings confirmed that growth arrest was caused by the loss of OsACBP6.

**Fig. 3:**
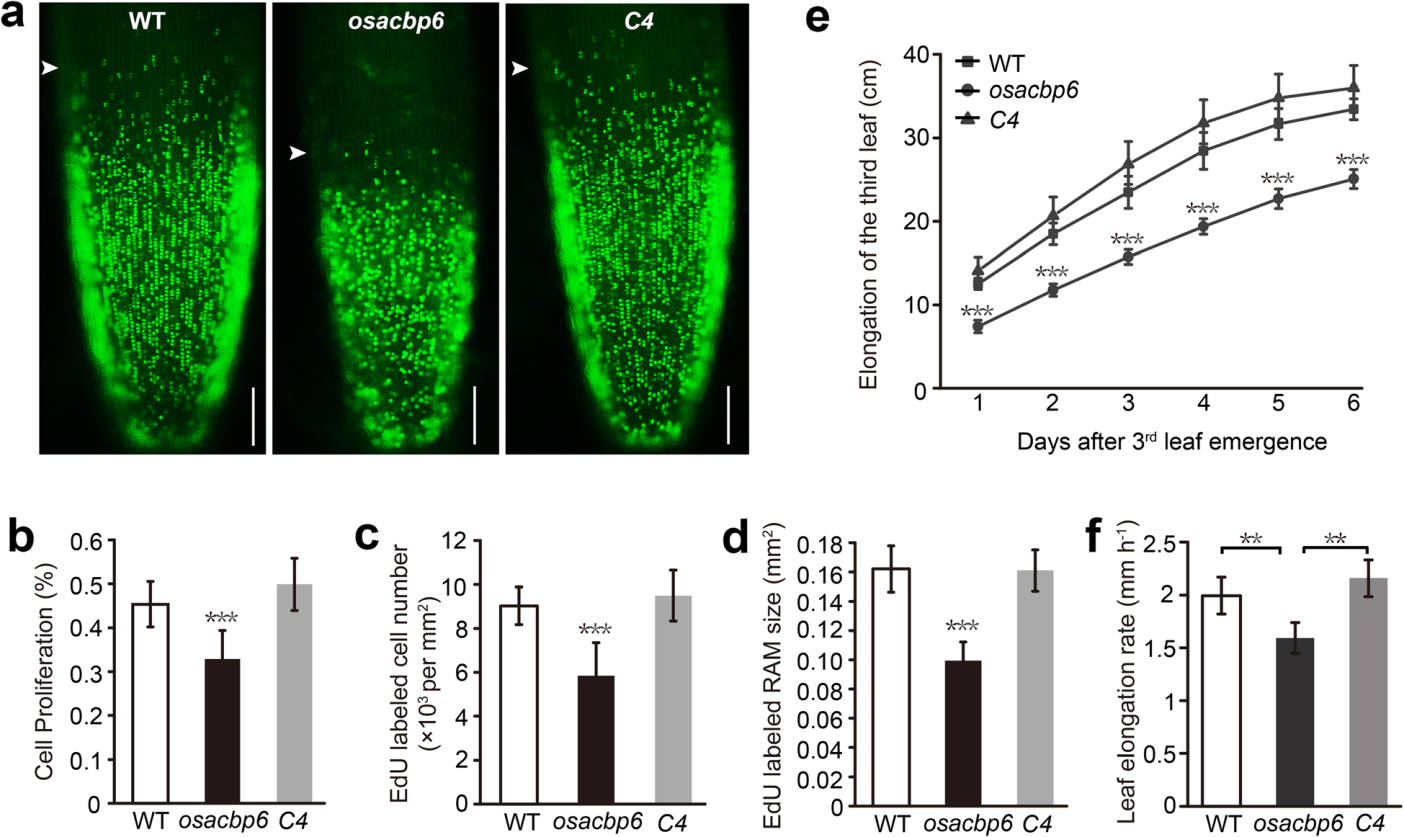
Reduced root meristem activity and leaf elongation in the rice *osacbp6* mutant. **a** Representative cell proliferation in the root tip cells of 5-day-old wild-type (WT), *osacbp6* and complemented line *osacbp6*-*C4* (*C4*) seedlings visualized by 5-ethynyl-2′-deoxyuridine (EdU) staining. Bars, 100 μm. Arrowheads indicate the border of EdU staining. **b** - **d** Relative quantification of the proliferating cells labeled with EdU, including the percentage of cell proliferation calculated as the ratio of EdU/DAPI (**b**), average proliferating cell number (**c**), and EdU-labeled root apical meristem size (**d**). Values are means ± SD (*n* = 10). **e** and **f** Measurement of the length of the third leaf (**e**) and leaf elongation rate (**f**). Values are means ± SD (*n* = 5). Asterisks indicate significant differences as evaluated by Student’s *t*-tests: **P < 0.01, ***P < 0.001. DAPI, 4′,6-Diamidino-2-Phenylindole; WT, *Oryza sativa* var. *japonica* cv. Dongjin

In addition, expression information from the Rice eFP Browser (http://bar.utoronto.ca/efprice/cgi-bin/efpWeb.cgi) showed that *OsACBP6* is highly expressed at seedling roots, young leaf and mature leaf (Fig. S4), suggesting OsACBP6 may have important roles in these tissues. Therefore, the root meristem activity was further investigated, 5-ethynyl-2′-deoxyuridine (EdU) staining indicated that reduced EdU labeling was observed in the root tip of *osacbp6* but not in the complemented line (Fig. 3a-d), indicating that cell proliferation in the root meristem affected by the loss of OsACBP6 could be complemented by the OsACBP6 ORF. On the other hand, observation of leaf growth showed that the emergence of the third leaf of *osacbp6* was one day later than the wild type. When the third leaf of *osacbp6* emerged from the leaf sheath, the third leaf length of the wild type and the complemented line was about 6 cm longer than *osacbp6* (Fig. 3e). Meanwhile, the leaf elongation rate of *osacbp6* was significantly lower than the wild type and the complemented line (Fig. 3f). These results indicated both root and the leaf development were affected in the *osacbp6* mutant and function was restored upon complementation.

### Changes in Acyl-CoA and Lipid Profiles in *osacbp6*

**Fig. 4:**
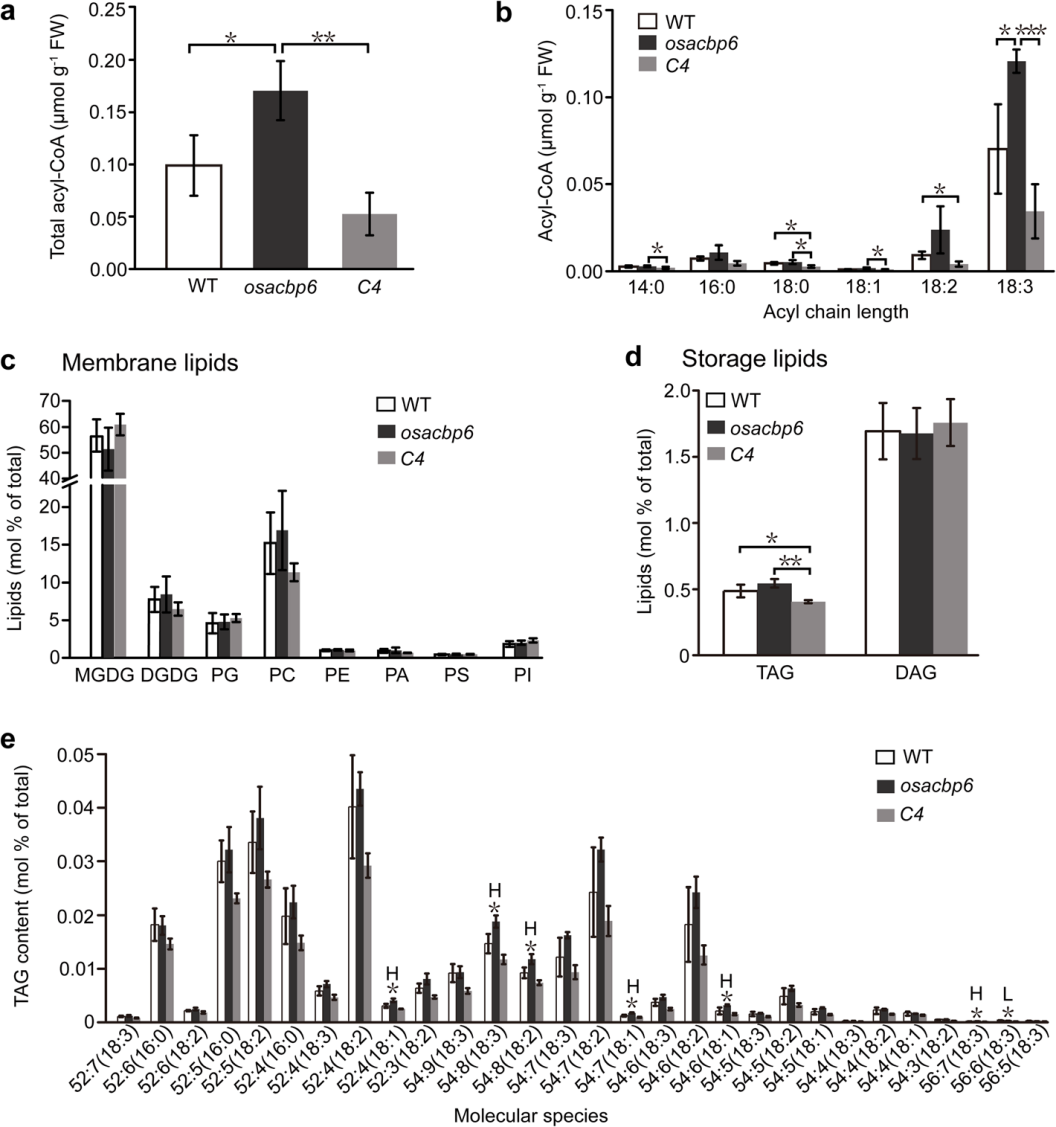
Acyl-coenzyme A (CoA) and lipid profiles of 21-day-old leaves. **a** Comparison of total acyl-CoA content of the wild type rice (WT), the *osacbp6* mutant and the complemented line *osacbp6-C4* (*C4*). **b** The concentration of different acyl-CoA species. **c** Membrane lipid content (mol % of total lipids analyzed). Values are means ± SD (*n* = 3). **d** Storage lipid content (mol % of total lipids analyzed). Values are means ± SD (*n* = 3). **e** The abundance of selected individual TAG molecular species (mol % of total) in 21-day-old leaves of the rice *osacbp6* mutant, WT, and *C4*. Values are means ± SD (*n* = 3). H, value of *osacbp6* is higher than WT; L, value of *osacbp6* is lower than WT. Values of *C4* are all significantly lower than *osacbp6* as evaluated by Student’s *t*-tests. Asterisks indicate significant differences as evaluated by Student’s *t*-tests: *P < 0.05, **P < 0.01, ***P < 0.001. FW, fresh weight; WT, *Oryza sativa* var. *japonica* cv. Dongjin. DAG, Diacylglycerol; TAG, Triacylglycerol; MGDG, Monogalactosyldiacylglycerol; DGDG, Digalactosyldiacylglycerol; PC, Phosphatidylcholine; PG, Phosphatidylglycerol; PA, Phosphatidic acid; PI, Phosphatidylinositol; PE, Phosphatidylethanolamine; PS, Phosphatidylserine

To gain an insight into the relationship between the loss of OsACBP6 and compromised growth, the acyl-CoA profiles in *osacbp6* was determined. The absence of OsACBP6 function is expected to disrupt fatty acid *β*-oxidation and result in the accumulation of long-chain acyl-CoAs. To test this hypothesis, a comparison was made amongst the acyl-CoA content in 21-day-old leaves of *osacbp6*, the wild type and the complemented line. As revealed in acyl-CoA measurements, the content of acyl-CoAs in *osacbp6* was significantly higher than the wild type and complemented line (*osacbp6-C4*) (Fig. 4a). Amongst all tested acyl-CoA esters, C18:3-CoA was notably enriched in the leaves of 21-day-old *osacbp6* in contrast to the wild type and *osacbp6-C4* (Fig. 4b). When compared with *osacbp6*, the level of C14:0-CoA, C18:0-CoA and C18:1-CoA showed a decrease in *osacbp6-C4* (Fig. 4b). In contrast to wild type, C18:2-CoA had significantly declined in *osacbp6-C4* while C16:0-CoA remained unchanged in all samples (Fig. 4b). These findings indicate that loss of OsACBP6 had likely compromised the maintenance of an acyl-CoA pool, particularly in the utilization of C18:3-CoA.

When the lipid composition of 21-day-old leaves was compared amongst *osacbp6,* the wild type, and the complemented line, few differences were apparent in all samples. The total amounts of membrane lipid species analyzed including monogalactosyldiacylglycerol (MGDG), digalactosyldiacylglycerol (DGDG), phosphatidylglycerol (PG), phosphatidylcholine (PC), PE, PA, phosphatidylserine (PS), and phosphatidylinositol (PI) remained unchanged (Fig. 4c, Fig. S5). On the other hand, in comparison with *osacbp6*, the total storage lipids TAG content significantly declined in *osacbp6-C4* (Fig. 4d, Dataset S1), although difference between the wild type and *osacbp6* was not significant. However, analysis of TAG molecular species revealed that TAG molecules 52:4(18:1), 54:8(18:3), 54:8(18:2), 54:7(18:1), 54:6(18:1), and 56:7(18:3) were significantly elevated in *osacbp6* in comparison with the wild type (Fig. 4e). In contrast to *osacbp6*, the complemented line showed significant decrease in TAG56 molecules 56:5(18:3), 56:6(18:3) and 56:7(18:3), most of the TAG54 molecules (54:9(18:3), 54:8(18:3), 54:8(18:2), 54:7(18:3), 54:7(18:2), 54:7(18:1), 54:6(18:3), 54:6(18:2), 54:6(18:1), 54:5(18:3), 54:5(18:2), 54:5(18:1), 54:4(18:3), 54:4(18:2), 54:4(18:1), 54:3(18:2)) and several TAG52 molecules (Dataset S1). Meanwhile, the complemented line when compared with the wild type, exhibited lower amounts of TAG molecules 52:7(18:3), 52:5(16:0), 52:3(18:3), 52:3(18:2), 54:9(18:3), 54:8(18:2), 54:6(18:3), 56:6(18:3), and 56:5(18:3) (Fig. 4e, Dataset S1).

### Comparative Transcriptomic Analysis between *osacbp6* and the Wild Type

To further analyze the potential role of OsACBP6 in growth regulation, a comparative transcriptomic analysis was performed between *osacbp6* and the wild type at different growth stages (Dataset S2, S3, S4, S5, S6, S7). Differentially expressed genes (DEGs) in *osacbp6* were associated with primary metabolic processes, including the light reaction, cell wall and lipid metabolism; and secondary metabolic processes related to terpenes, flavonoids, and phenylpropanoids. In *osacbp6*, genes involved in fatty acid synthesis and elongation, phospholipid synthesis, and lipid degradation appeared to be affected (Fig. S6). However, the key enzymes in *β*-oxidation, such as acyl-CoA oxidase, multifunctional protein, and 3-ketoacyl-CoA thiolase were not differentially expressed in *osacbp6* (Fig. S6). Furthermore, most of the DEGs were responsive to biotic stress (Fig. S7a). Compared with the wild type, various categories related to biotic stress were differentially expressed in *osacbp6*, including plant hormone signaling, PR proteins, cell wall, proteolysis, signaling, TFs, heat shock proteins, secondary metabolites. DEGs associated with defense hormone (ABA, JA, salicylic acid (SA), and ethylene) and growth hormone (auxin, gibberellin (GA), brassinosteroid (BR) and cytokinin) (Fig. S7b) and differentially expressed TFs (Fig. S7c) are displayed in Dataset S8 and S9, respectively. Amongst differentially expressed TFs, basic helix-loop-helix (bHLH) was strongly activated in the young seedlings, especially in 5-day-old roots (Fig. S7c). In 21-day-old leaves, WRKY was more activated than in the other samples (Fig. S7c).

**Fig. 5:**
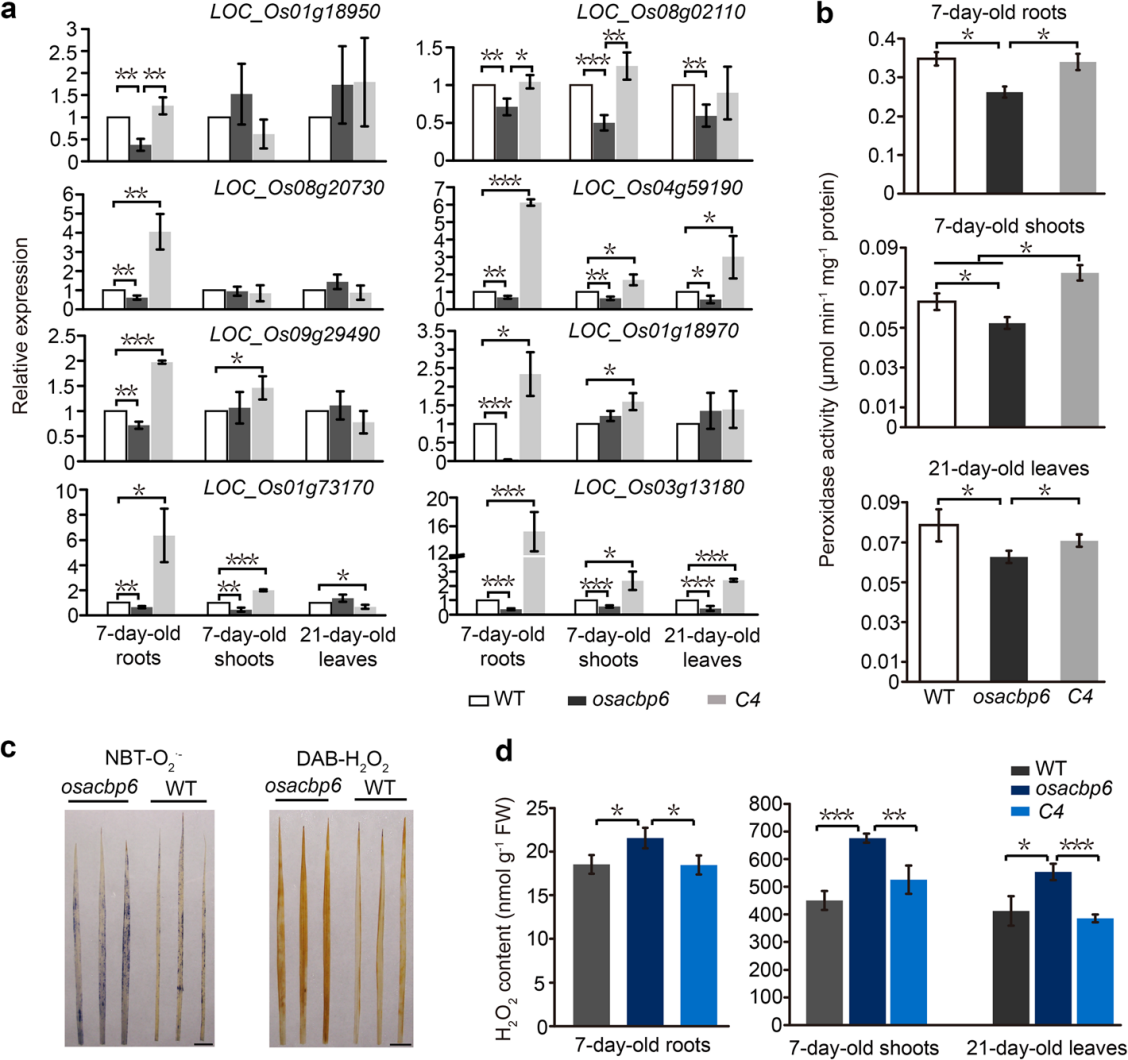
Disruption of reactive oxygen species homeostasis in the rice *osacbp6* mutant. **a** Relative expression of genes encoding class III peroxidases. Values are mean ± SD (*n* = 3). **b** Comparison of peroxidase activities of the roots and shoots of 7-day-old seedlings and leaves of 21-day-old wild type (WT), *osacbp6* and complemented line *osacbp6*-*C4* (*C4*). Values are means ± SD (*n* = 3). **c** Accumulation of superoxide anion (O_2_^∙-^) and hydrogen peroxide (H_2_O_2_) in the leaf blades of *osacbp6*. Leaf blades of 21-day-old WT and *osacbp6* were subjected to nitroblue tetrazolium (NBT) staining to detect O_2_^∙-^ or 3,3′-diaminobenzidine tetrahydrochloride (DAB) staining to detect H_2_O_2_ in the dark for 3 and 24 h, respectively. Bars, 1 cm. **d** H_2_O_2_ content in the roots and shoots of 7-day-old seedlings and leaves of 21-day-old WT, *osacbp6* and *C4*. Values are means ± SD (*n* = 3). Asterisks indicate significant differences as evaluated by Student’s *t*-tests: *P < 0.05, **P < 0.01, ***P < 0.001. WT, *Oryza sativa* var. *japonica* cv. Dongjin

Significantly enriched Gene Ontology (GO) categories were illustrated in Dataset S2. The GO term oxidation reduction was the most enriched in young seedlings and was common to all samples, except for the lack of a significantly enriched GO term in 7-day-old shoots. Another significantly enriched category was a response to oxidative stress that was only evident in the downregulated DEGs, and solely consisted of putative peroxidase precursor genes (class III peroxidase). Gene expression of peroxidases was verified by quantitative real-time RT-PCR (qRT-PCR). In comparison to the wild type, eight peroxidases were downregulated in 7-day-old *osacbp6* roots and upregulated in the complemented line (Fig. 5a). In 7-day-old shoots and 21-day-old leaves, the number of differentially expressed peroxidase was lower than 7-day-old roots. In particular, *LOC_Os03g13180* and *LOC_Os04g59190* had decreased in *osacbp6* in contrast to an increase in the complemented line in all test samples (Fig. 5a). In addition, expression information from the Rice eFP Browser (http://bar.utoronto.ca/efprice/cgi-bin/efpWeb.cgi) indicated that most of these eight peroxidases are highly expressed in roots (Fig. S8), suggesting probable significant roles in roots. Furthermore, the peroxidase activities were compared amongst *osacbp6*, the wild type, and the complemented line. In comparison with the wild type, the peroxidase activities in *osacbp6* decreased 25% in 7-day-old roots, 17% in 7-day-old shoots, and 17% in 21-day-old leaves (Fig. 5b). As expected, the level of peroxidase activities in the complemented line remained similar to the wild type with the exception of an increase in 7-day-old shoots (Fig. 5b). Given that peroxidases are bifunctional enzymes that can oxidize substrates at the expense of H_2_O_2_ but also produce ROS (Passardi et al. 2004), an abundant decline in the expression of peroxidases and reduced peroxidase activities suggest that ROS homeostasis in *osacbp6* is perturbed. Taken together, a loss in OsACBP6 had resulted in transcriptional changes in the expression of genes related to redox, H_2_O_2_ scavenging, and defense responses.

### ROS Homeostasis Is Perturbed in *osacbp6*

To examine whether the ROS levels were altered in *osacbp6*, superoxide anion (O_2_^∙-^) and H_2_O_2_ contents were examined. The blue and brown coloration in 21-day-old *osacbp6* leaf blades indicated the accumulation of O_2_^∙-^ and H_2_O_2_, respectively (Fig. 5c). Furthermore, H_2_O_2_ was quantified in *osacbp6*, the wild type, and the complemented line (Fig. 5d). The H_2_O_2_ levels in the root and shoot of 7-day-old *osacbp6* seedling and the leaves of 21-day-old *osacbp6* were significantly higher than the wild type and complemented line. Meanwhile, there was no significant difference between the wild type and complemented line suggesting the restoration of OsACBP6 function in the latter.

**Fig. 6:**
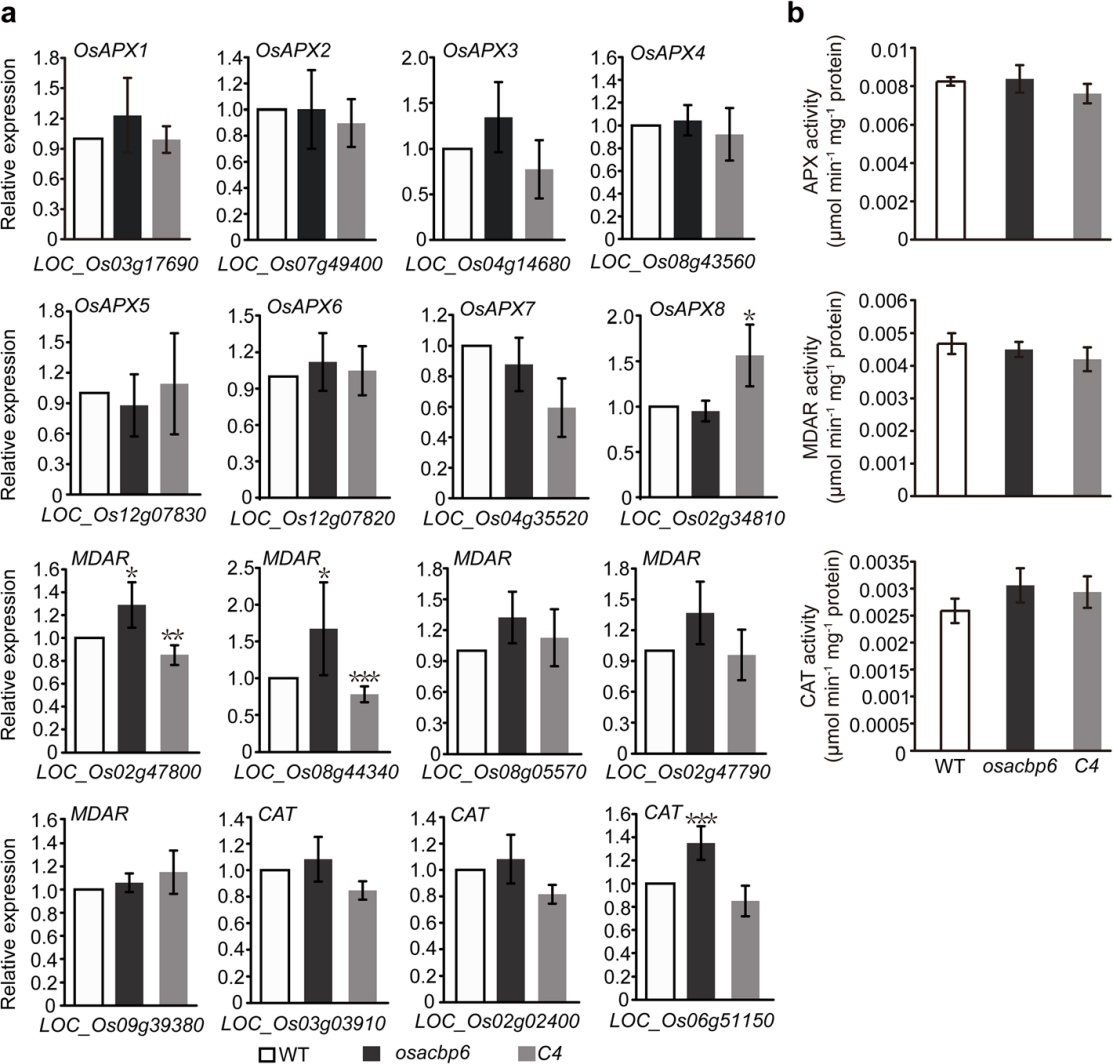
The expression levels and activities of rice genes associated with reactive oxygen species scavenging. **a** Relative expression in the leaves of 21-day-old wild type rice (WT), *osacbp6* and complemented lines *osacbp6*-*C4* (*C4*) were determined by quantitative real time RT-PCR (qRT-PCR). Values are means ± SD (*n* = 3). b Comparison of activities of APX, MDAR and CAT. Enzyme activity was detected from 21-day-old leaves of WT, *osacbp6* and *C4*. Values are means ± SD (*n* = 3). Asterisks indicate significant differences as evaluated by Student’s *t*-tests: *P < 0.05, **P < 0.01, ***P < 0.001. APX, ascorbate peroxidase; MDAR, monodehydroascorbate reductase; CAT, catalase; WT, *Oryza sativa* var. *japonica* cv. Dongjin

As OsACBP6 was localized at the peroxisomes (Meng et al. 2014), and H_2_O_2_ overaccumulated in *osacbp6*, peroxisomal ROS scavenging was next explored. Given that plant peroxisomes employ two major enzymatic antioxidant systems, catalase (CAT) and ascorbate peroxidase (APX)/monodehydroascorbate reductase (MDAR) system, to detoxify the H_2_O_2_ generated by *β*-oxidation (Eastmond 2007), the expression of *APX*, *MDAR*, and *CAT* was examined in *osacbp6*, the wild type and complemented line by qRT-PCR (Fig. 6). In comparison to the wild type, there was no significant difference in the expression of the eight *APX* genes in *osacbp6*, including peroxisomal localized *OsAPX3* and *OsAPX4* (Kaur and Hu 2011). The expression of five *MDAR* genes was examined. Except *LOC_Os08g05570*, the rest four *MDAR* genes are all peroxisomal localized (Kaur and Hu 2011). Two of the five *MDAR* genes was enhanced in *osacbp6*. In particular, *LOC_Os08g44340* was induced in *osacbp6* but repressed in the complemented line. Also, the expression of one of the three *CAT* genes was slightly elevated. Hence, despite H_2_O_2_ accumulation in *osacbp6*, the peroxisomal ROS scavenging system did not seem to be activated. Moreover, measurements of APX, MDAR and CAT activities further support that there was no significant difference in peroxisomal ROS scavenging system between *osacbp6* and the wild type (Fig. 6b). Above findings suggested that the distribution of H_2_O_2_ may not be confined to the peroxisome.

### Intrinsic JA Accumulated in *osacbp6*

**Fig. 7:**
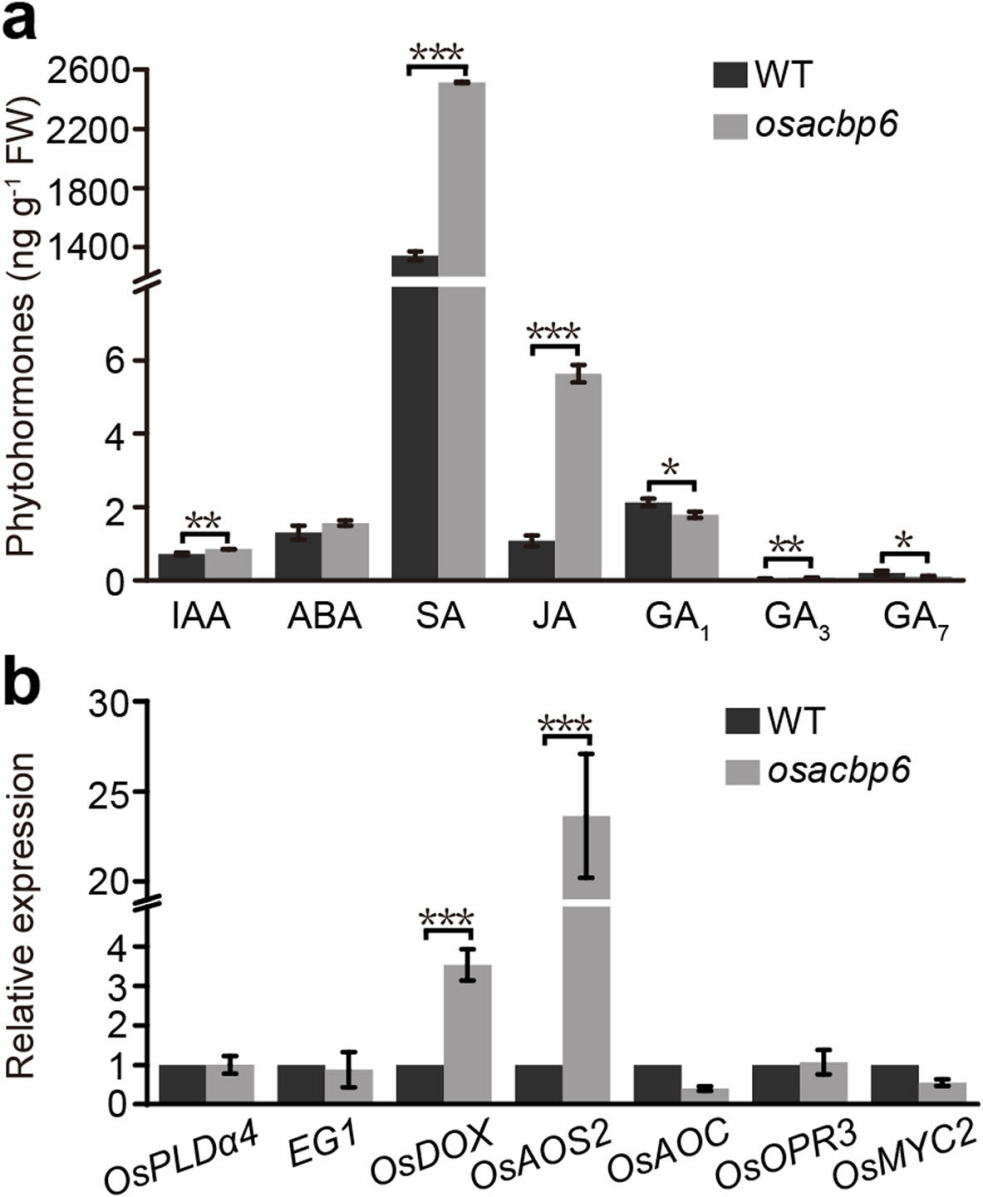
Alteration of plant hormone levels in the rice *osacbp6* mutant. **a** Measurement of plant hormone content in 21-day-old leaves. **b** Relative expression of genes related to JA biosynthesis and its signaling pathway. Values are means ± SD (*n* = 3). Asterisks indicate significant differences between the wild type (WT) and *osacbp6* as evaluated by Student’s *t*-tests: *P < 0.05, **P < 0.01, ***P < 0.001. WT, *Oryza sativa* var. *japonica* cv. Dongjin. IAA, indole-3-acetic acid; ABA, abscisic acid; SA, salicylic acid; JA, jasmonic acid; GA, gibberellin; PLD, phospholipase D; EG1, EXTRA GLUME 1; DOX, α-dioxygenases; AOS, allene oxide synthase; AOC, allene oxide cyclase; OPR, OPDA reductase

Arising from an observation in differentially expressed plant hormone-related genes, JA biosynthesis was investigated next to address if it was compromised in *osacbp6*. Because JA is produced through *β*-oxidation in the peroxisomes (Hu et al. 2012) and the overexpression of *OsACBP6* in the Arabidopsis *pxa1* mutant had rescued JA production after wound treatment (Meng et al. 2014). The intrinsic JA level was significantly elevated in 21-day-old *osacbp6* leaves (5.64 ng g^− 1^), five times higher than the wild type (1.08 ng g^− 1^) (Fig. 7a). Other plant hormones (SA, IAA and GA_3_) were also enhanced in *osacbp6* over the wild type (Fig. 7a). Therefore, measurements of hormone levels indicated that JA and SA production was not blocked in *osacbp6*.

Subsequently, the expression of genes related to JA biosynthesis and the JA signaling pathway in *osacbp6* were examined. JA biosynthesis includes the release of α-linolenic acid (α-LeA) from the lipid membrane by phospholipases, oxygenation of α-LeA by lipoxygenase or α-dioxygenases (DOXs) followed by steps catalyzed by allene oxide synthase (AOS) and allene oxide cyclase (AOC) to yield the JA precursor, *cis*-12-oxo-phytodienoic acid (OPDA). OPDA is then reduced by OPDA reductase (OPR) and subjected to *β*-oxidation to generate JA (Wasternack and Feussner 2018). When the expression of phospholipases D (*OsPLDα4*) and lipase EXTRA GLUME 1 (*EG1*) that function in releasing α-LeA (Qi et al. 2011; Cai et al. 2014), *OsDOX*, *OsAOS2*, *OsAOC*, *OsOPR3* (Koeduka et al. 2005; Mei et al. 2006; Guo et al. 2014) and transcription factor *OsMYC2* that positively regulates the JA-responsive genes (Uji et al. 2016), were examined, only *OsDOX* and *OsAOS2* expression increased in *osacbp6*, while others remained a similar level to the wild type (Fig. 7b), consistent with transcriptomic data. Given that *OsDOX* is activated by H_2_O_2_ (Zhu et al. 2013), elevated JA level in *osacbp6* would be a consequence of repressed H_2_O_2_ scavenging in *osacbp6*.

## Discussion

The level and availability of acyl-CoA is strictly controlled by proteins with binding abilities to meet the cellular requirement (Neess et al. 2015). Acyl-CoAs are sequestered and directed toward specific metabolic pathways (Neess et al. 2015). It has been proposed that OsACBP6 is implicated in the formation and regulation of the peroxisomal acyl-CoA pool and donation of acyl-CoA for *β*-oxidation (Meng et al. 2014). This study revealed that the disruption of peroxisomal matrix-localized *OsACBP6* in rice resulted in growth retardation in both roots and leaves as well as smaller grain size, resembling the phenotype of Arabidopsis and barley *β*-oxidation dysfunction mutants deficient in the peroxisomal ABC transporter (Zolman et al. 2001; Mendiondo et al. 2014), although the IBA sensitivity remained in *osacbp6*. The above findings suggest that *osacbp6* was not severely affected in *β*-oxidation function. However, increased acyl-CoA levels (specifically C18:3-CoA) and some TAG molecular species content observed in *osacbp6* over the wild type suggest it bears similarity to the Arabidopsis mutants defective in acyl-CoA import (peroxisomal ABC transporter *pxa1*/*cts*/*ped3*) and activation (peroxisomal long-chain acyl-CoA synthetase *lacs6lacs7*) and core enzymes of fatty acid *β*-oxidation (*mfp2*, *kat2*) (Germain et al. 2001; Fulda et al. 2004; Rylott et al. 2006; Graham 2008). The functions of plant peroxisomal ABC transporters are highly conserved between eudicot Arabidopsis and monocot barley (Zolman et al. 2001; Mendiondo et al. 2014). Although the role of the rice peroxisomal ABC transporter is yet unclear, two orthologues of Arabidopsis PXA1 co-exists (Kaur and Hu 2011). Localization of OsACBP6 in the peroxisomal matrix suggests that OsACBP6 would not be able to transfer acyl-CoAs directly from the cytosol into the peroxisome, although OsACBP6 does have a putative *N*-terminal transmembrane domain. Taken together with findings that recombinant OsACBP6 binds C18:2-CoA and C18:3-CoA with high binding affinities in vitro (Meng et al. 2011), the increased acyl-CoA levels in *osacbp6* and their corresponding decline in the complemented line, support a role for OsACBP6 in the peroxisome most likely associated in the maintenance of a peroxisomal acyl-CoA pool, especially for C18:3-CoA, and to facilitate efficient utilization of acyl-CoAs imported by PXA1 orthologues for subsequent *β*-oxidation. Loss of OsACBP6 might disrupt the utilization of C18:3-CoA in the following *β*-oxidation and induce a feedback inhibition to the import of C18:3-CoA into the peroxisomes which might lead to the accumulation of C18:3-CoA in the cytosol. On the other hand, DEG-related to fatty acids synthesis, phospholipids biosynthesis, and TAG biosynthesis were limited in *osacbp6*, suggesting loss of OsACBP6 may not increase TAG content. However, it has been proposed that acyl-CoA utilization has a feedback influence on the rate of TAG lipolysis, and the mechanism of acyl-CoA-mediated inhibition of TAG breakdown is not clear (Graham 2008; Quettier and Eastmond 2009). Therefore, in *osacbp6*, the relationship between acyl-CoA accumulation and an increase in some TAG molecular species content awaits to be further elucidated.

Arabidopsis mutants defective in *β*-oxidation-related genes commonly display failure in early seedling establishment due to a block in TAG turnover (Graham 2008). The phenotypic difference between the wild type and these mutants is indistinguishable at the vegetative phase but some of them show abnormal inflorescence later (Richmond and Bleecker 1999; Fulda et al. 2004; Rylott et al. 2006; Wiszniewski et al. 2014). In contrast, as starch in the endosperm is the major energy supply for monocots post-germinative growth, the seedling establishment does not seem to be affected by a deficiency in fatty acid *β*-oxidation (Mendiondo et al. 2014; Xu et al. 2017). However, distinguishable phenotypes emerge at the vegetative phase. A rice *aim1* mutant appeared as a short root mutant with reduced meristem activity due to blocked SA biosynthesis and reduced ROS level (Xu et al. 2017). By repressing the expression of redox and ROS-scavenging genes and maintaining a proper level of ROS, SA maintains root meristem activity (Xu et al. 2017). In this study, the root meristem activity of *osacbp6* also declined, but unlike *aim1*, its H_2_O_2_ level in root was elevated. It has been demonstrated that Arabidopsis bHLH TF UPBEAT1 (UPB1) regulates the balance of root tip cell proliferation and differentiation by controlling the expression of a set of peroxidases and therefore the balance of O_2_^∙-^ and H_2_O_2_ (Tsukagoshi et al. 2010). Ectopic expression of *UPB1* represses the peroxidases and results in H_2_O_2_ accumulation and decreased meristem size in the root tip (Tsukagoshi et al. 2010). In *osacbp6* root, reduction in root meristem activity, repression in gene expression of a set of peroxidases, decrease in peroxidase activities, and accumulation of H_2_O_2_ occurred, which were restored in the complemented lines. These findings are partially consistent with ROS-controlled root growth regulated by UPB1 (Tsukagoshi et al. 2010). Unfortunately, as a UPB1 orthologue has not been identified in the rice genome using a BLAST search in NCBI (National Center for Biotechnology Information, http://blast.ncbi.nlm.nih.gov/Blast.cgi), the TF responsible for such regulation remains elusive.

The regulatory mechanisms for the growth of leaf and root are known to be different (Lu et al. 2014). In Arabidopsis, leaf growth and final size are controlled by MYB-like TF KUODA1 (KUA1)-regulated peroxidase activities which in turn maintain ROS homeostasis (Lu et al. 2014). Although leaf and root growth are controlled by different TFs, the direct targets of these TFs are peroxidases and by regulation in the expression of peroxidases, ROS homeostasis is maintained (Tsukagoshi et al. 2010; Lu et al. 2014). In *osacbp6*, compromised leaf development was represented by slow elongation and accompanied by a high H_2_O_2_ level. Consistent with root, leaf peroxidases of *osacbp6* were also differentially expressed and activities were reduced. In addition, given that O_2_^∙-^ can be converted to H_2_O_2_ spontaneously or by enzymatic activities (Apel and Hirt 2004), the elevated level of O_2_^∙-^ in *osacbp6* also might be an alternative source of H_2_O_2_ accumulation. Due to the complexity of peroxidases in promoting or restricting plant growth (Passardi et al. 2004), the regulation mechanism of the reduced peroxidase activities and accumulation of ROS in retarded leaf development of *osacbp6* were elusive. They might contribute to abnormal cell proliferation or differentiation and ultimately reduce leaf development. Taken together, from the findings on root and leaf, retarded growth in *osacbp6* mostly possible resulted from a disruption of ROS homeostasis. On the other hand, as a higher level of H_2_O_2_ occurred throughout *osacbp6* development, it appears that stress response is constitutively activated in *osacbp6*. Diminished growth of *osacbp6* is consistent with studies on growth-defense tradeoffs, in which plant growth is inhibited by the activation of defense responses (Guo et al. 2018). Meanwhile, the examination of various plant hormones further supports this hypothesis. Alteration in the plant hormone levels was noted in *osacbp6*, especially an increase in JA content. The core jasmonate signaling pathway functions in the perception of various developmental and stress-related cues and mediation of transcriptional responses (Howe et al. 2018). One of the output responses of the jasmonate signaling pathway is developmental responses, including growth inhibition, defensive structures, and effects on reproduction and fertility (Howe et al. 2018). As the first step of JA biosynthesis, plants employ different lipases to liberate α-LeA from chloroplast membrane lipids, including DONGLE (DGL), DEFECTIVE IN ANTHER DEHISCENCE1 (DAD1), and phospholipase A (PLA) PLA-Iγ1 in Arabidopsis, PLDα4 and α5 and EG1 in rice (Ellinger et al. 2010; Qi et al. 2011; Cai et al. 2014). In contrast to an induction triggered in JA biosynthesis (Qi et al. 2011), PLDα4 and α5 showed no change in *osacbp6*. Despite that EG1 is mainly required for development in rice (Cai et al. 2014), there was no change for EG1 in *osacbp6*. Limited alteration in expression of these lipases is consistent with lack in an obvious loss of chloroplast membrane lipids in comparison to the wild type, suggesting α-LeA in *osacbp6* is sufficient to produce enough JA to confer the balance between growth and defense. Amongst enzymes involved in JA biosynthesis, only DOX and AOS were induced in *osacbp6*. Given that DOX is activated by H_2_O_2_ (Zhu et al. 2013), enhanced of JA biosynthesis in *osacbp6* under normal growth conditions appears to be a perception of the elevated H_2_O_2_ level and in turn adjusted the corresponding transcriptional responses to diminish growth. Elevated JA level in *osacbp6* indicated that JA production from the *β*-oxidation of JA precursor, OPDA, was not blocked due to the loss of OsACBP6. As we discussed above, OsACBP6 would not be responsible for direct import of substrate into the peroxisomes. Therefore, in combination with the changes in the expression of genes related to JA biosynthesis, elevated JA level is most likely resulted from the secondary effects of the loss of OsACBP6.

Acyl-CoA esters are not only important intermediates in lipid metabolism but also play a role in regulating multiple cellular processes (Schmidt et al. 2018; Lung and Chye 2019). Recently, direct evidence showed that acyl-CoA esters function in the cellular signaling pathway in Arabidopsis (Schmidt et al. 2018). An increased C18:1-CoA level promoted the dissociation of TF RELATED TO APETALA 2.12 (RAP2.12) with its partner AtACBP1, which determined subsequent hypoxia-specific gene expression (Schmidt et al. 2018). Meanwhile, C16:0-CoA, C18:0-CoA, and C18:1-CoA triggered distinct transcriptional responses (Schmidt et al. 2018). Our findings that elevated acyl-CoA content in *osacbp6* was accompanied by alterations in lipid composition as well as ROS and plant hormone levels further support the critical roles of acyl-CoA esters in the signaling network in both dicots and monocots. Although OsACBP6 has two cytosolic orthologues (AtACBP4 and AtACBP5) in Arabidopsis, a mere single mutation in any made them indistinguishable from the wild type (Hsiao et al. 2015). Even in the *acbp4acbp5* double mutant, the phenotypic discrepancy was limited because another cytosolic AtACBP6 co-exists (Hsiao et al. 2015). Based on our results, a mutation in OsACBP6 led to phenotypic and physiological changes in rice. Furthermore, the correlation of increased acyl-CoA pool size with the loss of OsACBP6 also suggests a possible role of overexpression of OsACBP6 in the wild type in facilitating acyl-CoAs utilization.

## Conclusions

In conclusion, acyl-CoA profiling in this study revealed the role of ACBPs in acyl-CoA utilization is conserved in both Arabidopsis and rice. However, it is worth mentioning that growth retardation in *osacbp6* was associated with a series of changes including changes in acyl-CoA pool size, lipid metabolism, ROS content, and plant hormone levels as well as other transcriptional response, indicating the correlation of enhanced acyl-CoAs content with defense responses.

## Methods

### Plant Materials and Growth Conditions

T-DNA line PFG_1B-14,906 (designated as *osacbp6*) was obtained from Postech PFG T-DNA, South Korea (Ryu et al. 2004). To prepare the samples for transcriptomic analysis, seeds of wild-type rice (*Oryza sativa* var. *japonica* cv. Dongjin) and *osacbp6* were soaked in water and germinated in dark at 25 °C for 2 days. To collect the roots and shoots, germinated seeds grown in water were placed in the growth chamber under a 10-h-light (30 °C) / 14-h-dark (28 °C) cycle. Roots and shoots were sampled separately at 5 and 7 days after imbibition. To collect the leaf blade, germinated seeds were grown in soil under the same growth condition as described above. The leaf blades were collected at 21 days after imbibition. For IBA treatment, germinated seeds were hydroponically cultured in Hoagland solution supplemented with 10 mg L^− 1^ IBA for 7 days. Cell proliferation in the root meristem was visualized by the incorporation of EdU into DNA at S-phase (Kotogany et al. 2010). EdU staining was performed using an EdU kit (C10310, Apollo 488, Ribobio) according to Li et al. (2015). Briefly, roots of 5-day-old rice seedlings (10 seedlings for each genotype) were immersed in a 50 μM EdU solution for 2 h. After fixation in 4% paraformaldehyde for 30 min, roots were incubated with freshly-prepared Apollo solution for 30 min, followed by 4′,6-Diamidino-2-Phenylindole (DAPI, 1 mg mL^− 1^) staining for 10 min and fluorescence microscopy. Images were analyzed using ImageJ software (Schneider et al. 2012). In order to observe leaf elongation, the length of the third leaf was measured daily with a ruler using the top edge of the pot as a reference (Pettko-Szandtner et al. 2015). Measurements were carried out on five plants starting from the emergence of the third leaf for 6 days.

### T-DNA Confirmation

The gene-specific primer (ML2067) and T-DNA left border primer (2717-LB) were used in PCR to confirm the T-DNA insertion in PFG_1B-14,906 (*osacbp6*). The combinations of gene-specific primer pair ML2066/ML2067 and 2717-LB/ML2067 were used to screen for the homozygote mutant. Gene-specific primer pair ML1050 and ML1051 was used in semiquantitative RT-PCR to detect *OsACBP6* expression. The primer sequences were ML2066: 5′-ATCAGGGCTGAGGTGCTA-3′; ML2067: 5′-AGGGCTTCAGAATCGTATGG-3′; 2717-LB: 5′-ACGCTGAACTTGTGGCCGTT-3′; ML1050: 5′-CCAGATCTTCCCGCTTCCAGAACGAC-3′; and ML1051: 5′-CTGAATTCTTAAGTCATGCCCTCACTG-3′.

### Generation of *osacbp6-*Complemented Transgenic Rice

An *OsACBP6* putative promoter region containing 2.0-kb upstream of the transcription start site was amplified using PCR with primer pair Promoter-F (5′-CAGTCGTCTCACAACTGCCACTGTAAATTTGTCTA-3′ with the *Bsm*BI underlined) and Promoter-R (5′-CAGTCGTCTCAGCTTCGCGGGCGATGAATGA-3′ with *Bsm*BI underlined). The 1.97-kb open reading frame (ORF) of *OsACBP6* was amplified by RT-PCR using primer pair ORF-F (5′-CAGTCGTCTCAAAGCATGGCGAGCTCCGGACTCGC-3′ with *Bsm*BI underlined) and ORF-R (5′-CAGTCGTCTCATACATCAAGACTCGGACTTATCAG-3′ with *Bsm*BI underlined). To construct the *OsACBP6* promoter driving *OsACBP6* cDNA plasmid (*pOsACBP6::OsACBP6*) for complementation, the PCR products of promoter and ORF were digested by *Bsm*BI, and the binary vector pBWA(V)H-ccdb-Tnos was digested by *Eco*31I. The promoter and the ORF regions were ligated to the vector pBWA(V)H-ccdb-Tnos using T4 ligase. The *pOsACBP6::OsACBP6* construct was then introduced into *osacbp6* by *Agrobacterium*-mediated transformation. Positive transgenic rice lines were confirmed by PCR and hygromycin screening, and the T3 generation was used to observe changes in the phenotype.

### Acyl-CoA and Lipid Profiling

For acyl-CoA measurement, acyl-CoA esters were extracted from freshly harvested leaves from at least five individual 21-day-old (100 mg, three replicates) soil-grown plants according to Woldegiorgis et al. (1985) and Larson and Graham (2001). For lipid profiling, leaves from at least five individual 21-day-old soil-grown plants were freshly collected for analysis (60 mg, three replicates). Plant materials were inactivated with hot isopropanol, and total lipid extraction was performed as previously described (Welti et al. 2002).

Lipid and acyl-CoA measurements were performed on Exion UPLC coupled with QTRAP 6500 PLUS (SCIEX) under the conditions: curtain gas = 20 psi, ion spray voltage = 5500 V, temperature = 400 °C, ion source gas 1 = 35 psi, and ion source gas 2 = 35 psi. Polar lipid and glycerol lipids (diacylglycerols (DAGs) and TAGs) were analyzed as previously described (Lam et al. 2014; Gao et al. 2017). Separation of galactolipids MGDG and DGDG were achieved according to Cheong et al. (2014) and Gao et al. (2017).

### Transcriptomic Analysis and DEG Identification

Total RNA from 5-day-old roots, 5-day-old shoots, 7-day-old roots, 7-day-old shoots and 21-day-old leaves, respectively, was extracted using the RNeasy Plant Mini Kit (Qiagen). Transcriptome analysis was conducted at Annoroad Gene Technology Corporation (Beijing, China). The data from three replicates were collected and analyzed. Genes whose expression is increased by over 100% or decreased by over 50% with adjusted *P*-value ≤0.05 were considered as DEGs.

### Gene Ontology Enrichment and MapMan Analysis

GO enrichment was performed using AgriGO v2.0 (Tian et al. 2017) (http://systemsbiology.cau.edu.cn/agriGOv2/). Under the species “*Oryza sativa japonica*”, singular enrichment analysis was performed. GO terms in biological process with FDR value < 0.005 were considered as significant and analyzed further. The biological processes and metabolic pathways were systematically evaluated using MapMan analysis (Thimm et al. 2004), MapMan software version 3.6.0RC1 (https://mapman.gabipd.org/) was employed. The rice gene identifier (osa_MSU_v7_2017-09-05_mapping.txt) that was imported to MapMan was downloaded from GoMapMan (Ramsak et al. 2014) (http://www.gomapman.org/export/current/mapman). The “Metabolism overview” and the “Cellular response” sections of MapMan were used to display the changes in DEGs with putative functions.

### Quantitative Real-Time RT-PCR

Total RNA from 7-day-old roots, 7-day-old shoots and 21-day-old leaves, respectively, was extracted using the RNeasy Plant Mini Kit (Qiagen) and reverse transcribed by ReverTra Ace® qPCR RT Master Mix with gDNA Remover (Toyobo). qRT-PCR was performed on Light-Cycler Roche 480 (Roche) with LightCycler® 480 SYBR Green I Master (Roche) under the following conditions: 95 °C for 10 min, then 40 cycles of 95 °C for 10 s and 60 °C for 20 s. Primer sequences were listed in Dataset S10. *ACTIN1* and *UBIQUITIN1* were used as reference genes. The relative expression level was calculated using the 2^-ΔΔCt^ method (Schmittgen and Livak 2008).

### Detection of ROS and Quantitative Measurement of Hydrogen Peroxide (H_2_O_2_)

Nitroblue tetrazolium (NBT) and 3,3′-diaminobenzidine tetrahydrochloride (DAB) staining were conducted as described by Fukao et al. (2011). Each leaf blade from a 21-day-old rice plant grown in soil was immersed in a solution containing 0.5 mg mL^− 1^ NBT (dissolved in 10 mM potassium phosphate buffer, pH 7.6) and 1 mg mL^− 1^ DAB (dissolved in 50 mM Tris-Acetate buffer, pH 5.0). After incubation under vacuum for 5 min, samples were kept in the dark at 25 °C for 3 h for NBT staining and 24 h for DAB staining. Subsequently, plant materials were boiled in 95% (v/v) ethanol for 20 min to remove chlorophyll and rehydrated in 40% (v/v) glycerol. Each experiment was repeated on at least five plants and representative images are displayed.

H_2_O_2_ content was measured using the Amplex Red Hydrogen Peroxide/Peroxidase Assay Kit (Invitrogen). Briefly, roots and shoots of 7-day-old seedlings and 21-day-old leaf blades from at least three individual plants were freshly collected (50 mg, three replicates) and ground to a fine powder in liquid nitrogen followed by the immediate addition of 1 mL ice-cold 20 mM sodium phosphate buffer (pH 6.5). The supernatant was collected by centrifugation (10,000 *g*, 4 °C, 10 min) followed by analysis according to the manufacturer’s instructions.

### Measurement of Antioxidative Enzyme Activity

Peroxidase activity was measured according to Chen et al. (2013). Briefly, roots and shoots of 7-day-old seedlings and 21-day-old leaf blades from at least six individual plants were freshly collected and ground in 50 mM sodium phosphate buffer (pH 7.8) supplemented with 1% polyvinylpyrrolidone and *β*-mercaptoethanol (1:5, m/v) on ice. The supernatant was collected after centrifugation (13,000 *g*, 4 °C, 15 min) and used for peroxidase activity assay. The reaction mixture (2 mL) composed of 50 mM sodium acetate buffer (pH 5.6), 5.4 mM guaiacol, 15 mM H_2_O_2_, and 100 μL of enzyme extract. The absorbance at 470 nm increase in 1 min was recorded using Cary 60 UV-Vis (Agilent). Peroxidase activity was determined by the increase in the absorbance at 470 nm per minute per mg protein. The enzyme extract from 21-day-old leaf blades was also used for determination of CAT, APX and MDAR activity. CAT and APX activity were measured according to Wu and Yang (2016). The reaction mixture for CAT activity determination composed of composed of 50 mM sodium phosphate buffer (pH 7.8) 1.5 mL, ddH_2_O 1 mL, enzyme extract 200 μL and 300 μL 0.1 M H_2_O_2_. The reaction mixture for APX activity determination composed of 50 mM sodium phosphate buffer (pH 7.8) 1.25 mL, 5 mM ascorbic acid (AsA) 200 μL, enzyme extract 50 μL and 500 μL 0.1 mM H_2_O_2_. While MDAR activity was measured according to Hossain and Asada (1985). The reaction mixture composed of 50 mM sodium phosphate buffer (pH 7.8) 450 μL, 0.1 mM AsA 500 μL, 0.1 mM NADH 800 μL, enzyme extract 100 μL and 0.14 unit of ascorbate oxidase. CAT, APX and MDAR activity were determined by the decrease in the absorbance at 240 nm, 290 nm and 340 nm, respectively, per minute per mg protein.

### Quantification of Plant Hormones

Plant hormone extraction and quantification were conducted according to Pan et al. (2010) with minor modifications. Briefly, leaf tissue was ground into fine powder in liquid nitrogen. Plant hormones were extracted from 1 g powder with 10 mL isopropanol/H_2_O/concentrated HCl (2:1:0.002, v/v/v) extraction buffer supplemented with 8 μL internal standards solution containing 1 μg mL^− 1^ of each internal standard compound (d_5_-IAA, d_6_-ABA, d_6_-SA, H_2_JA, and d_2_-GA_4_). After shaking for 30 min at 4 °C, 20 mL dichloromethane was added to the extraction buffer followed by further shaking for 30 min at 4 °C. Samples were then centrifuged at 13000 *g* for 5 min and the lower phase was transferred, concentrated to dryness with nitrogen flow in the dark and resolved with 400 μL methanol (0.1% formic acid). Hormones were quantified using HPLC-MS/MS (SCIEX 6500 QTRAP) equipped with Poroshell 120 SB-C18 column (Agilent).

## Supplementary Information

**Additional file 1: Fig. S1** Growth of the rice *osacbp6* mutant and the wild type (WT) in soil. **a** Images representing 5- and 7-day-old seedlings grown in soil. Bars, 1 cm. **b** Shoot and primary root length comparison between WT and *osacbp6* (left) and primary root reduction of *osacbp6* (right) Values are means ± SD (*n* = 13). Asterisks indicate significant differences between WT and *osacbp6* as evaluated by Student’s *t*-tests: ***P < 0.001. WT, *Oryza sativa* var. *japonica* cv. Dongjin.

**Additional file 2: Fig. S2** Grain size and indole-3-butyric acid (IBA) sensitivity of *osacbp6*. **a** The grain length of *osacbp6* is shorter than WT. Values are means ± SD (*n* = 30). Asterisks indicate significant differences between WT and *osacbp6* as evaluated by Student’s *t*-tests: ***P < 0.001. **b** *osacbp6* is sensitive to the growth inhibition effect of IBA. Values are means ± SD (*n* = 5). Asterisks indicate significant differences between mock and IBA treatment as evaluated by Student’s *t*-tests: ***P < 0.001. WT, *Oryza sativa* var. *japonica* cv. Dongjin.

**Additional file 3: Fig. S3** Recovery in leaf and grain length of the rice *osacbp6* mutant by complementation of *OsACBP6*. **a** Relative expression of *OsACBP6* in the 7-day-old seedlings from the wild type (WT), *osacbp6*, complemented lines *osacbp6*-*C1* (*C1*), *osacbp6*-*C2* (*C2*) and *osacbp6*-*C4* (*C4*) measured by quantitative real time RT-PCR (qRT-PCR) using *OsACBP6* specific primer pair ML1113 and ML1114. Values are means ± SD (*n* = 3). **b** and **c** Leaf length of 21-day-old soil-grown seedlings (**b**) and grain size (**c**) are compared amongst *osacbp6*, WT, and the complemented lines *C1*, *C2* and *C4*. Values are mean ± SD (leaf length: *n* = 5; grain size: *n* = 30). L, value of *osacbp6* lower than WT and the complemented lines. H, value of the complemented line higher than WT. Asterisks indicate significant differences as evaluated by Student’s *t*-tests: *P < 0.05, **P < 0.01, ***P < 0.001. WT, *Oryza sativa* var. *japonica* cv. Dongjin.

**Additional file 4: Fig. S4** Expression of *OsACBP6* from the Rice eFP Browser. Absolute expression levels in the seedling root, young leaf and mature leaf were retrieved from the Rice eFP Browser (http://bar.utoronto.ca/efprice/cgi-bin/efpWeb.cgi).

**Additional file 5: Fig. S5** Membrane lipid content (mol % of total) in 21-day-old leaves. Values are means ± SD (*n* = 3). WT, wild type; *C4*, complemented line *osacbp6*-*C4*; PC, Phosphatidylcholine; PE, Phosphatidylethanolamine; PI, Phosphatidylinositol; PS, Phosphatidylserine; PA, Phosphatidic acid; MGDG, Monogalactosyldiacylglycerol; DGDG, Digalactosyldiacylglycerol; PG, Phosphatidylglycerol.

**Additional file 6: Fig. S6** Differential expression of genes involved in lipid metabolism in *osacbp6*. **a**-**c** Expression of genes associated with fatty acid synthesis and elongation (**a**) phospholipid synthesis (**b**), and lipid degradation (**c**), respectively. Values are mean of Log_2_ fold change (*n* = 3). The color in each cell represents the expression level based on the Log_2_ fold change. Cells without color indicate the genes were not differentially expressed. R5, 5-day-old roots; R7, 7-day-old roots; S5, 5-day-old shoots; S7, 7-day-old shoots; L21, 21-day-old leaves. KCS, beta-ketoacyl-CoA synthase; ACSL, long-chain acyl-CoA synthetase; FAD, omega-3 fatty acid desaturase; GPAT, glycerol 3-phosphate acyltransferase; CFA, cyclopropane-fatty-acyl-phospholipid synthase; GDSL, GDSL-like lipase; SDR, short-chain dehydrogenases/reductase.

**Additional file 7: Fig. S7** Analysis of differentially expressed genes related to cellular response and regulation in *osacbp6*. **a** Numbers of differentially expressed genes at different stages and organs. **b** and **c** Heat map of phytohormones (**b**) and selected transcription factors (**c**) in *osacbp6* based on the MapMan results, respectively. Each square represents a differentially expressed gene. The color indicates the level of up-regulation (red) and down-regulation (blue) based on the value of mean Log_2_ fold change (*n* = 3). The full list of differentially expressed genes is displayed in Dataset S8 and S9. BR, brassinosteroid; GA, gibberellin; ABA, abscisic acid; JA, jasmonic acid; SA, salicylic acid; AP2/EREBP, APETALA2/ethylene-responsive element binding protein family; bZIP, basic leucine zipper transcription factor family; *MYB,* myeloblastosis transcription factor family; WRKY, WRKY transcription factor family; ABI3/VP1, ABA-INSENSITIVE3/VIVIPAROUSl transcription factor family; bHLH, basic helix-loop-helix transcription factor family; Aux/IAA, auxin/indole-3-acetic acid transcription factor family; NAC, NO APICAL MERISTEM (NAM), ARABIDOPSIS TRANSCRIPTION ACTIVATION FACTOR1–2 (ATAF1/2), and CUP-SHAPED COTYLEDON2 (CUC2) transcription factor family.

**Additional file 8: Fig. S8** Expression of peroxidases from the Rice eFP Browser. Absolute expression levels in the seedling root, young leaf, and mature leaf were retrieved from the Rice eFP Browser (http://bar.utoronto.ca/efprice/cgi-bin/efpWeb.cgi).

**Additional file 9.** Dataset S1-S10.

## Data Availability

The datasets supporting the conclusions of this article are included within the article and its additional files.
