# Supplementary figures and images for: RICE ACYL-COA-BINDING PROTEIN6 Affects Acyl-CoA Homeostasis and Growth in Rice
Source: Rice (N Y). 2020 Nov 6;13:75. doi: 10.1186/s12284-020-00435-y (PMC7647982; doi:10.1186/s12284-020-00435-y)

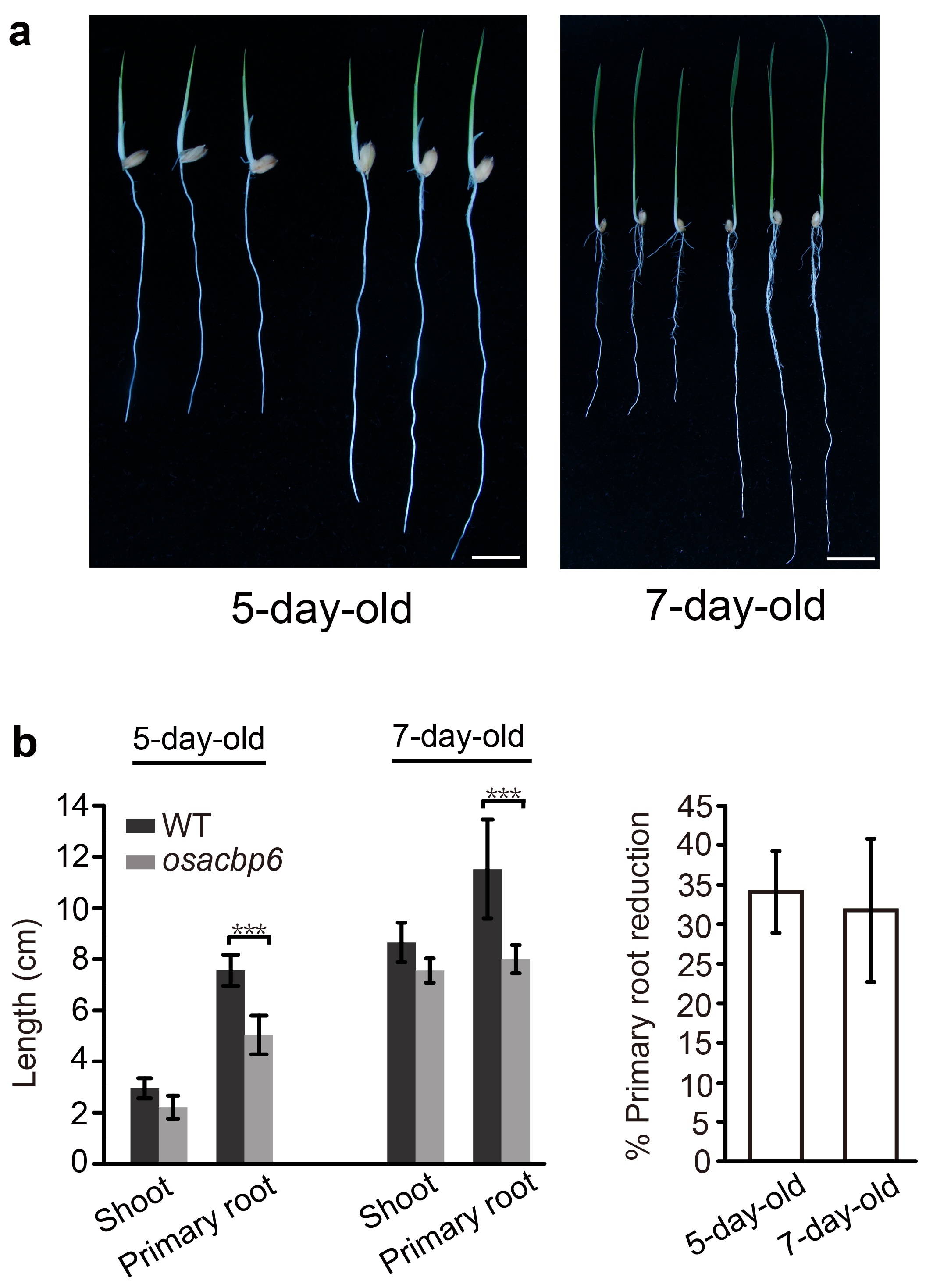

Supplement: Supplementary file 1 — Additional file 1: Fig. S1 Growth of the rice osacbp6 mutant and the wild type (WT) in soil. a Images representing 5- and 7-day-old seedlings grown in soil. Bars, 1 cm. b Shoot and primary root length comparison between WT and osacbp6 (left) and primary root reduction of osacbp6 (right) Values are means ± SD (n = 13). Asterisks indicate significant differences between WT and osacbp6 as evaluated by Student’s t-tests: ***P < 0.001. WT, Oryza sativa var. japonica cv. Dongjin. [file 12284_2020_435_MOESM1_ESM.tif]

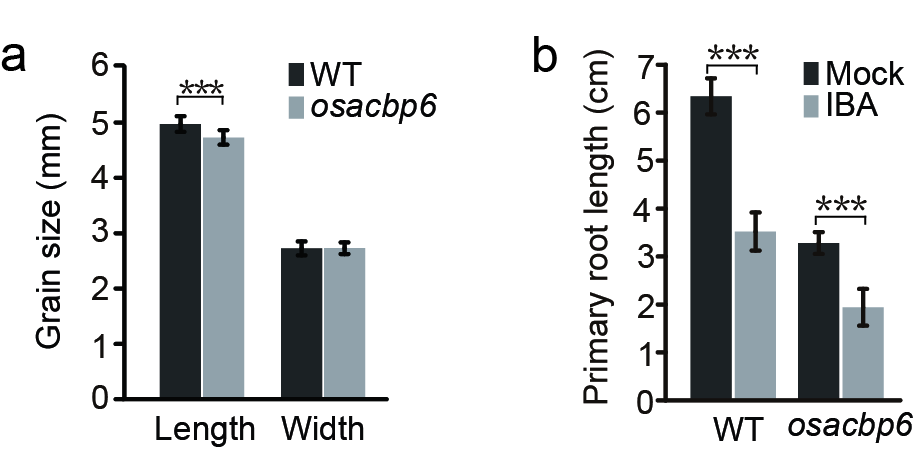

Supplement: Supplementary file 2 — Additional file 2: Fig. S2 Grain size and indole-3-butyric acid (IBA) sensitivity of osacbp6. a The grain length of osacbp6 is shorter than WT. Values are means ± SD (n = 30). Asterisks indicate significant differences between WT and osacbp6 as evaluated by Student’s t-tests: ***P < 0.001. b osacbp6 is sensitive to the growth inhibition effect of IBA. Values are means ± SD (n = 5). Asterisks indicate significant differences between mock and IBA treatment as evaluated by Student’s t-tests: ***P < 0.001. WT, Oryza sativa var. japonica cv. Dongjin. [file 12284_2020_435_MOESM2_ESM.tif]

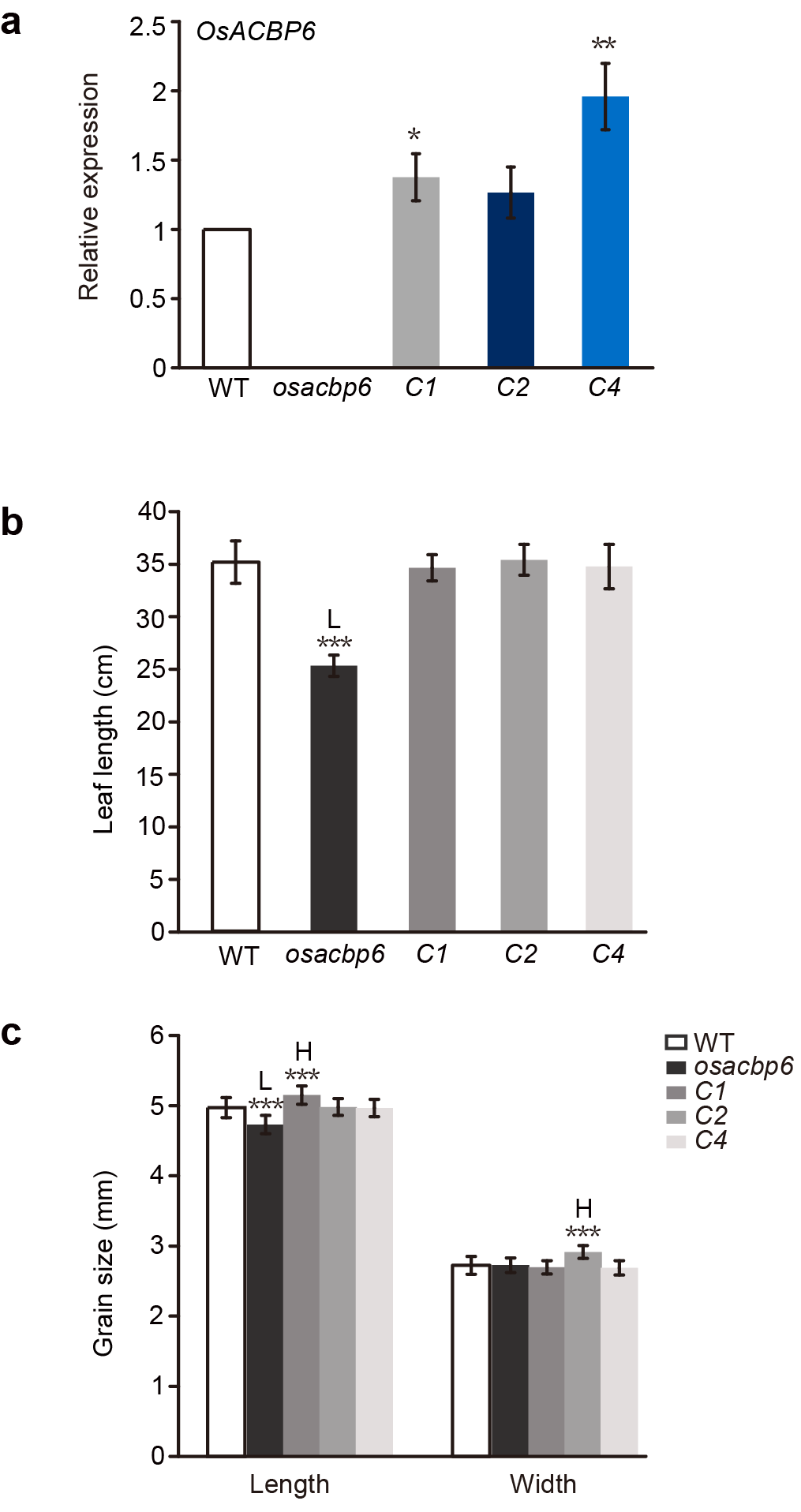

Supplement: Supplementary file 3 — Additional file 3: Fig. S3 Recovery in leaf and grain length of the rice osacbp6 mutant by complementation of OsACBP6. a Relative expression of OsACBP6 in the 7-day-old seedlings from the wild type (WT), osacbp6, complemented lines osacbp6-C1 (C1), osacbp6-C2 (C2) and osacbp6-C4 (C4) measured by quantitative real time RT-PCR (qRT-PCR) using OsACBP6 specific primer pair ML1113 and ML1114. Values are means ± SD (n = 3). b and c Leaf length of 21-day-old soil-grown seedlings (b) and grain size (c) are compared amongst osacbp6, WT, and the complemented lines C1, C2 and C4. Values are mean ± SD (leaf length: n = 5; grain size: n = 30). L, value of osacbp6 lower than WT and the complemented lines. H, value of the complemented line higher than WT. Asterisks indicate significant differences as evaluated by Student’s t-tests: *P < 0.05, **P < 0.01, ***P < 0.001. WT, Oryza sativa var. japonica cv. Dongjin. [file 12284_2020_435_MOESM3_ESM.tif]

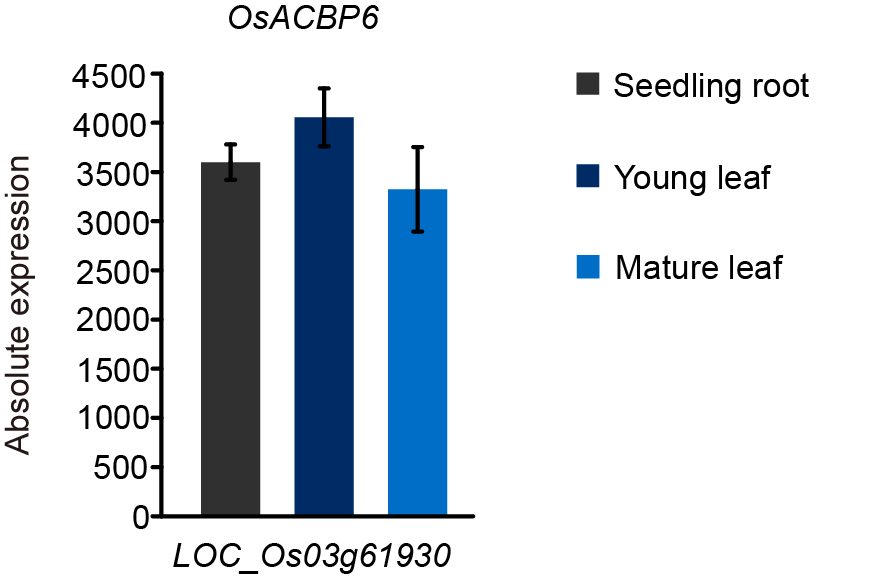

Supplement: Supplementary file 4 — Additional file 4: Fig. S4 Expression of OsACBP6 from the Rice eFP Browser. Absolute expression levels in the seedling root, young leaf and mature leaf were retrieved from the Rice eFP Browser (http://bar.utoronto.ca/efprice/cgi-bin/efpWeb.cgi). [file 12284_2020_435_MOESM4_ESM.tif]

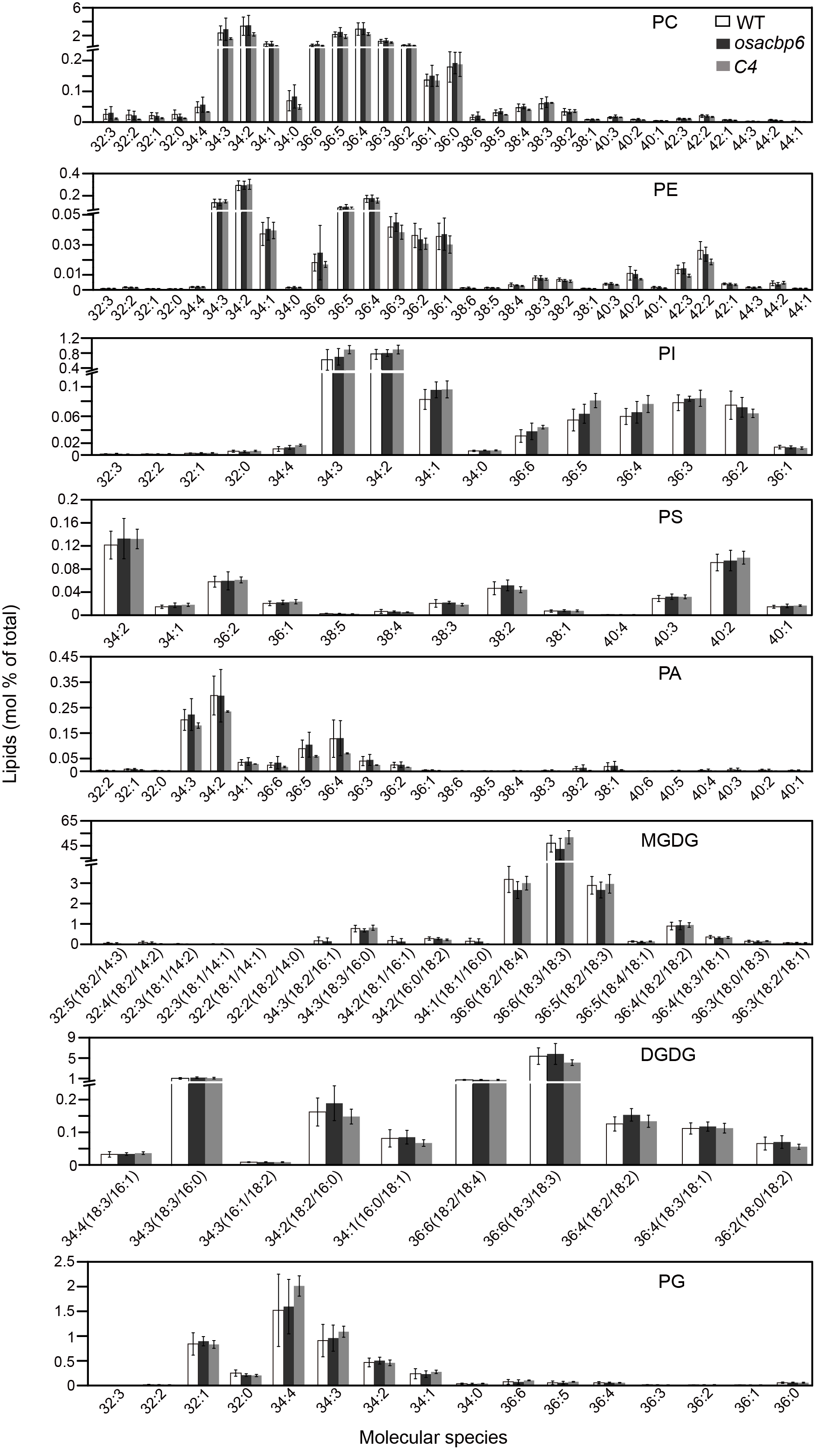

Supplement: Supplementary file 5 — Additional file 5: Fig. S5 Membrane lipid content (mol % of total) in 21-day-old leaves. Values are means ± SD (n = 3). WT, wild type; C4, complemented line osacbp6-C4; PC, Phosphatidylcholine; PE, Phosphatidylethanolamine; PI, Phosphatidylinositol; PS, Phosphatidylserine; PA, Phosphatidic acid; MGDG, Monogalactosyldiacylglycerol; DGDG, Digalactosyldiacylglycerol; PG, Phosphatidylglycerol. [file 12284_2020_435_MOESM5_ESM.tif]

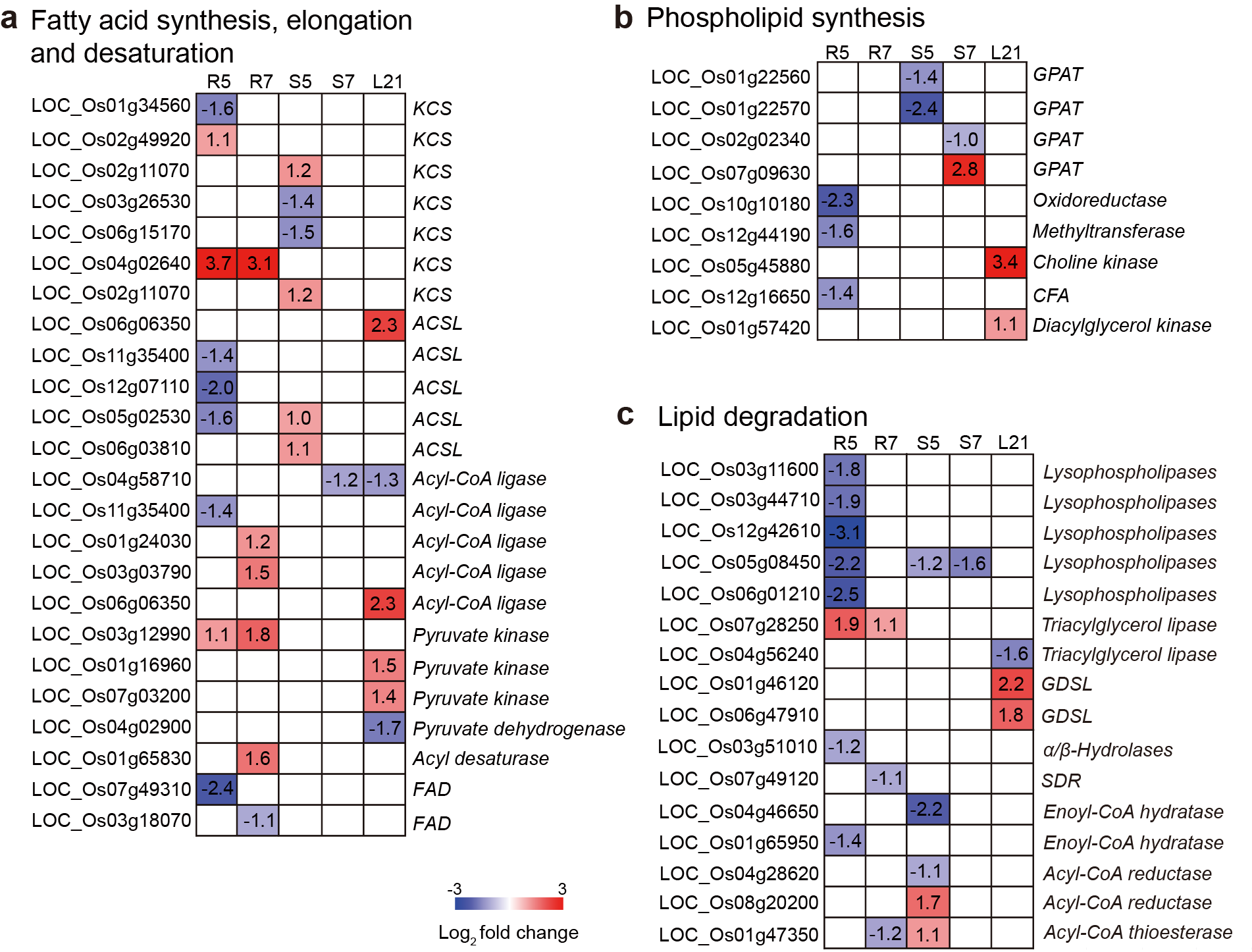

Supplement: Supplementary file 6 — Additional file 6: Fig. S6 Differential expression of genes involved in lipid metabolism in osacbp6. a-c Expression of genes associated with fatty acid synthesis and elongation (a) phospholipid synthesis (b), and lipid degradation (c), respectively. Values are mean of Log2 fold change (n = 3). The color in each cell represents the expression level based on the Log2 fold change. Cells without color indicate the genes were not differentially expressed. R5, 5-day-old roots; R7, 7-day-old roots; S5, 5-day-old shoots; S7, 7-day-old shoots; L21, 21-day-old leaves. KCS, beta-ketoacyl-CoA synthase; ACSL, long-chain acyl-CoA synthetase; FAD, omega-3 fatty acid desaturase; GPAT, glycerol 3-phosphate acyltransferase; CFA, cyclopropane-fatty-acyl-phospholipid synthase; GDSL, GDSL-like lipase; SDR, short-chain dehydrogenases/reductase. [file 12284_2020_435_MOESM6_ESM.tif]

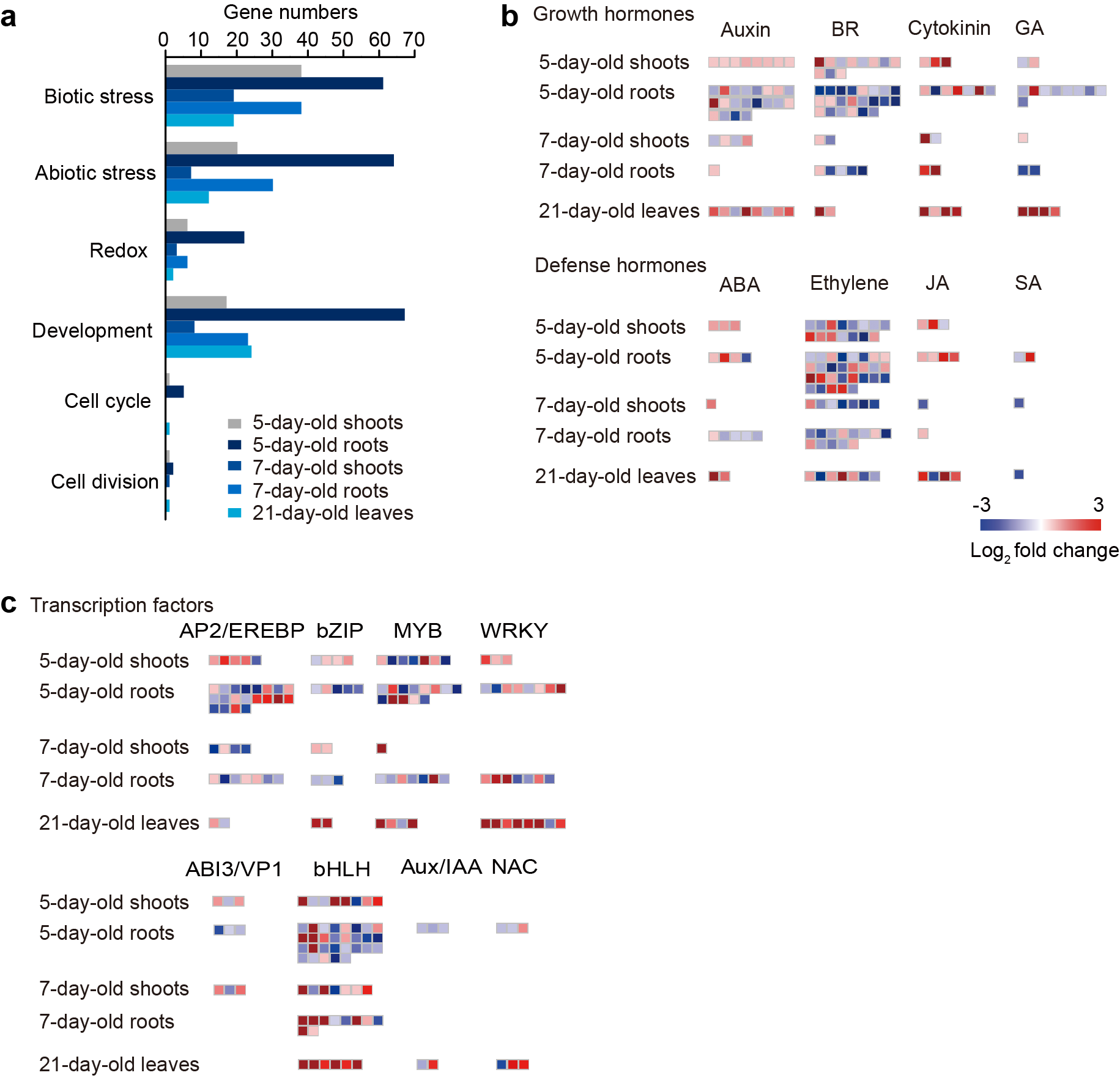

Supplement: Supplementary file 7 — Additional file 7: Fig. S7 Analysis of differentially expressed genes related to cellular response and regulation in osacbp6. a Numbers of differentially expressed genes at different stages and organs. b and c Heat map of phytohormones (b) and selected transcription factors (c) in osacbp6 based on the MapMan results, respectively. Each square represents a differentially expressed gene. The color indicates the level of up-regulation (red) and down-regulation (blue) based on the value of mean Log2 fold change (n = 3). The full list of differentially expressed genes is displayed in Dataset S8 and S9. BR, brassinosteroid; GA, gibberellin; ABA, abscisic acid; JA, jasmonic acid; SA, salicylic acid; AP2/EREBP, APETALA2/ethylene-responsive element binding protein family; bZIP, basic leucine zipper transcription factor family; MYB, myeloblastosis transcription factor family; WRKY, WRKY transcription factor family; ABI3/VP1, ABA-INSENSITIVE3/VIVIPAROUSl transcription factor family; bHLH, basic helix-loop-helix transcription factor family; Aux/IAA, auxin/indole-3-acetic acid transcription factor family; NAC, NO APICAL MERISTEM (NAM), ARABIDOPSIS TRANSCRIPTION ACTIVATION FACTOR1–2 (ATAF1/2), and CUP-SHAPED COTYLEDON2 (CUC2) transcription factor family. [file 12284_2020_435_MOESM7_ESM.tif]

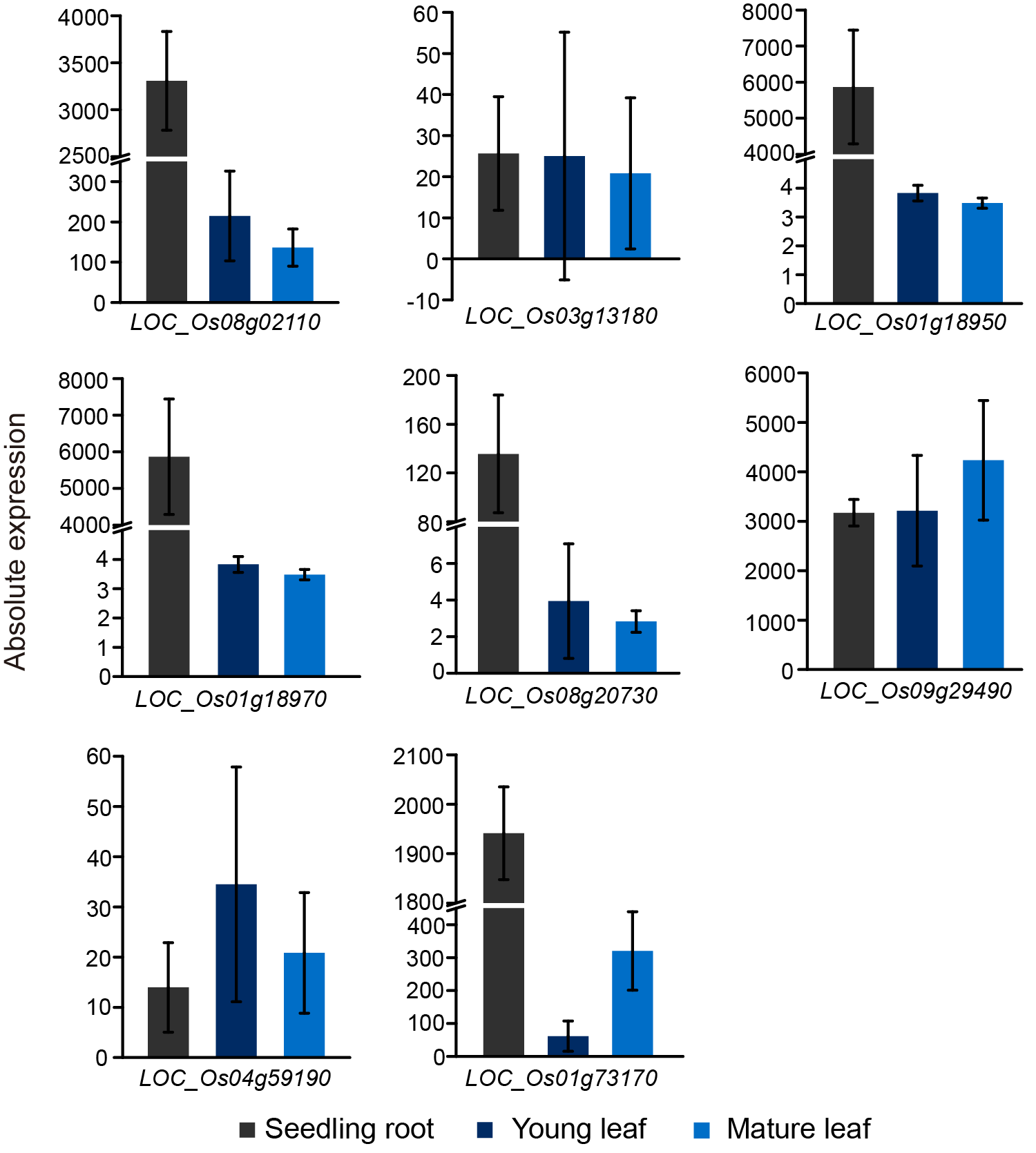

Supplement: Supplementary file 8 — Additional file 8: Fig. S8 Expression of peroxidases from the Rice eFP Browser. Absolute expression levels in the seedling root, young leaf, and mature leaf were retrieved from the Rice eFP Browser (http://bar.utoronto.ca/efprice/cgi-bin/efpWeb.cgi). [file 12284_2020_435_MOESM8_ESM.tif]
